# Signatures of selection in mammalian clock genes with coding trinucleotide repeats: Implications for studying the genomics of high‐pace adaptation

**DOI:** 10.1002/ece3.3223

**Published:** 2017-08-08

**Authors:** Melanie B. Prentice, Jeff Bowman, Jillian L. Lalor, Michelle M. McKay, Lindsay A. Thomson, Cristen M. Watt, Andrew G. McAdam, Dennis L. Murray, Paul J. Wilson

**Affiliations:** ^1^ Department of Environmental and Life Sciences Trent University Peterborough ON Canada; ^2^ Wildlife Research and Monitoring Section Ontario Ministry of Natural Resources and Forestry Peterborough ON Canada; ^3^ Biology Department Trent University Peterborough ON Canada; ^4^ Department of Integrative Biology University of Guelph Guelph ON Canada

**Keywords:** clock genes, coding trinucleotide repeats, contemporary adaptation, natural selection

## Abstract

Climate change is predicted to affect the reproductive ecology of wildlife; however, we have yet to understand if and how species can adapt to the rapid pace of change. Clock genes are functional genes likely critical for adaptation to shifting seasonal conditions through shifts in timing cues. Many of these genes contain coding trinucleotide repeats, which offer the potential for higher rates of change than single nucleotide polymorphisms (SNPs) at coding sites, and, thus, may translate to faster rates of adaptation in changing environments. We characterized repeats in 22 clock genes across all annotated mammal species and evaluated the potential for selection on repeat motifs in three clock genes (*NR1D1*,*CLOCK*, and *PER1*) in three congeneric species pairs with different latitudinal range limits: Canada lynx and bobcat (*Lynx canadensis* and *L. rufus*), northern and southern flying squirrels (*Glaucomys sabrinus* and *G. volans*), and white‐footed and deer mouse (*Peromyscus leucopus* and *P. maniculatus*). Signatures of positive selection were found in both the interspecific comparison of Canada lynx and bobcat, and intraspecific analyses in Canada lynx. Northern and southern flying squirrels showed differing frequencies at common *CLOCK* alleles and a signature of balancing selection. Regional excess homozygosity was found in the deer mouse at *PER1* suggesting disruptive selection, and further analyses suggested balancing selection in the white‐footed mouse. These preliminary signatures of selection and the presence of trinucleotide repeats within many clock genes warrant further consideration of the importance of candidate gene motifs for adaptation to climate change.

## 1 INTRODUCTION

The rapid pace of climate change is expected to profoundly alter the future phenology, range distribution, and physiology of wildlife (Bellard, Berteksmeier, Leadley, Thuiller, & Courchamp, 2012), with impacts on reproduction being of particular importance (Milligan, Holt, & Lloyd, 2009). A critical emerging question is whether species can evolve new seasonal strategies (Boutin & Lane, 2014; Merilä & Hendry, 2014). The underlying basis for this question lies in whether sufficient standing genetic variation or sufficient rates of molecular evolution (Barrett & Schluter, 2008; Hedrick, 2013) occur at key genes to keep pace with climate change. Ultimately, the rate of adaptive evolution in relation to the rate of climate change will contribute to the demographic effects of climate change on organisms and ecosystems (Bradshaw & Holzapfel, 2010; Bronson, 2009). This makes the characterization of adaptive genetic variation critical, in order to allow for a better understanding of the evolutionary potential and responses of species to environmental stressors (e.g., Harrisson, Paylova, Telonis‐Scott, & Sunnucks, 2014). Further, understanding how standing genetic variation and genomic elements operating at higher rates of change contribute to adaptability will be important to estimate the relative roles of genetics, plasticity, and epigenetics in defining the “response capacity” or “adaptive potential” of species. Mammals are a particular taxonomic group whose vulnerability to climate change may be underestimated (Schloss, Nuñez, & Lawler, 2012), and as a result, there is a recognized need to identify and characterize mammalian genes responding to climate change (Dawson, Jackson, House, Prentice, & Mace, 2011; Franks & Hoffmann, 2012).

The seasonal timing of life‐history events is often under the influence of selection as such events are frequently influenced by environmental cues (O'Malley, Ford, & Hard, 2010). Individuals that can anticipate the optimal timing of season‐specific activities (e.g., migration, reproduction) are predicted to demonstrate higher fitness, as they are able to exploit the most favorable resources throughout the year. Photoperiod is one such environmental cue that is often used to determine the optimal timing of life‐history strategies in species occupying seasonal environments (Bradshaw & Holzapfel, 2008). Species respond to photoperiod cues via their circadian clocks, molecular oscillators that sense and respond to changes in photoperiod by triggering various effects including hormone secretions in mammals (Goldman, 2001). In fact, the negative relationship observed between day length and amplitude of the circadian pacemaker may be the cause of latitudinal clines often observed in the timing of seasonal events of many species (Pittendrigh, Kyner, & Takamura, 1991). Heritability of photoperiod responsiveness has been observed in mammals (Bronson, 2009; Heideman, Bruno, Singley, & Smedley, 1999; Lynch, Heath, & Johnston, 1981), particularly at higher latitudes where dependence on photoperiod increases with variance in day length and cues circannual seasonal changes (Bradshaw & Holzapfel, 2010). Thus, it has been argued that climate change is likely to introduce significant reproductive challenges for species inhabiting higher latitudes that rely on photoperiod to cue breeding, because at such latitudes an uncoupling of the phase relationship between environmental conditions and photoperiodic cues can occur (Milligan et al., 2009). Further, as range redistributions proceed due to shifts in temperature, species may be exposed to novel photoperiods. As species can track shifts in temperature through range redistributions, their persistence will more critically require the adjustment of photoperiod responses rather than thermal tolerance (Bradshaw & Holzapfel, 2006). Clock genes are thus one category of functional genes likely critical for adaptation to shifting seasonal conditions and novel environments (Kondratova, Dubrovsky, Antoch, & Kondratov, 2010).

The candidate gene approach has been used empirically to identify patterns of adaptive genetic variation and disentangle such patterns from neutral genetic population structure (DeFaveri, Jonsson, & Merilä, 2013; Hemmer‐Hansen, Nielsen, Frydenberg, & Loeschke, 2007; Limborg et al., 2012; O'Malley et al., 2010). Candidate genes are selected based on known physiological functions perceived to be of relevance to the study species. This approach is supported where highly divergent allele frequencies are found, more often in genes with functions related to adaptive processes potentially under selection. Such genes are dissimilar to neutral regions of the genome, which are not expected to vary among populations experiencing high rates of gene flow. Here, we use the candidate gene approach to examine specific motifs in targeted functional genes, specifically coding trinucleotide repeats.

Coding trinucleotide repeats (cTNRs; e.g., polyQ = polyglutamine) are repeat structures that often are found in exonic regions of the genome and consist of units that are three nucleotides long due to selection against frame‐shift mutations, which would alter the reading frame of the transcribed protein (Duitama et al., 2014). Such repeat structures have traditionally been linked to human genetic diseases (e.g., Huntington's disease; MacDonald et al., 1993); however, an increasing number of studies now show that these motifs have a critical role in “normal” protein function and evolutionary adaptation (Haerty & Golding, 2010). Further, emerging studies on cTNRs indicate that these structures can have functional roles that are under selection (Bradshaw & Holzapfel, 2010; Haerty & Golding, 2010; Li, Liu, Wu, & Chen, 2012; Molla, Delcher, Sunyaev, Cantor, & Kasif, 2009; Mularoni, Ledda, Toll‐Riera, & Mar Albà, 2010) in addition to high levels of population variation that exert continuous and discrete functional phenotypes (Gemayel, Cho, Boeynaems, & Verstrepen, 2012; Gemayel, Vinces, Legendre, & Verstrepen, 2010; Kashi & King, 2006).

The mutational mechanism of cTNR structures has been associated with the purity of the repeat structure itself, where purer repeats are more likely to undergo further slippage (Kruglyak, Durrett, Schug, & Aquadro, 1998). This may be of adaptive value by generating phenotypic variation upon which selection can act (Kashi & King, 2006; Laidlaw et al., 2007). These repeats also offer the potential for high mutation rates (Gemayel et al., 2010, 2012), allowing for the rapid generation of novel alleles on the scale of contemporary adaptive evolution. This is particularly important in genes required for adaptation to climate change, in addition to a reliance on plasticity, regulatory elements (Bozek et al., 2009), and epigenetic effects (Ripperger & Merrow, 2011).

A number of recent latitudinal studies have examined the potential evolutionary and adaptive importance of cTNRs embedded in clock genes. For example, intraspecific studies have demonstrated correlations between repeat number of the *CLOCK* cTNR and latitude in birds (Johnsen et al., 2007) and fish (O'Malley et al., 2010) in addition to variation corresponding to earlier egg laying (Liedvogel, Szulkin, Knowles, Wood, & Sheldon, 2009). Further, the involvement of clock genes in seasonal entrainment has been demonstrated in mammals (Hazlerigg, Ebling, & Johnston, 2005), suggesting that cTNRs within these genes may play a role in seasonally fine‐tuning the circadian characteristics of species inhabiting higher latitudes. Collectively, these results suggest that environmental factors correlated with latitude (e.g., photoperiod) may be driving selection at cTNRs within clock genes that are critical for the seasonal adaptation of life‐history strategies. Thus, the characterization of cTNR structures in a range of other vertebrate species offers the potential to use the properties of microsatellite repeats (Press, Carlson, & Queitsch, 2014) to understand the genomics of adaptation.

Coding trinucleotide repeats have been observed in clock genes that facilitate the regulation of reproductive timing and social behaviors (Johnsen et al., 2007; Liedvogel & Sheldon, 2010; Liedvogel et al., 2009), genes associated with neuroprocesses (Whan et al., 2010), developmental homeobox genes, and transcription factors (Mularoni et al., 2010). For closely related species, their higher rates of mutation propose a mechanism for the convergence of pole‐ward allele sizes following climate‐induced range expansion. This is highly relevant to mammals, as many closely related species have evolved in complex and often isolated refugium patterns north or south of ice sheets during the Pleistocene (e.g., Shafer, Cullingham, Côté, & Coltman, 2010), thus allowing for the evolution of allelic repeat motifs specific to differential climatic conditions within an otherwise presumably conserved gene sequence.

Our objectives for this study were twofold. First, as wild mammal species have been infrequently characterized at clock genes for the presence of cTNR motifs, we wanted to characterize cTNRs within several candidate clock genes in a wide range of mammal species. Second, to evaluate the potential of clock genes for adaptation to differential latitudes, we compared three north–south congeneric species pairs at a selection of clock genes to determine the prevalence of cTNR repeats and levels of polymorphism. Due to the importance of such genes for circadian and circannual rhythms of mammal species, we hypothesized that clock genes are under selection in mammal species occurring along latitudinal clines. To test our hypothesis, we compared closely related species pairs adapted to different climatic niches and separated along latitudinal gradients at varying spatial scales. If clock genes are under selection in our study species, we expect to observe at least one of the following: clines in allele frequencies within or between species pairs, differentiation of allele frequencies between species pairs, departures from Hardy–Weinberg equilibrium (HWE), divergent patterns of differentiation (*F*_ST_) between neutral microsatellites and each candidate cTNR, and/or identification of our cTNR loci as outliers in comparison with neutral genetic population structure.

## 2 METHODS

### 2.1 Characterization of candidate clock genes in mammal species

Before testing for selection, we wanted to characterize the presence and abundance of cTNRs in 22 candidate clock genes of mammal species. We selected the genes *AANAT*,* ARNTL*,* ARNTL2*,* CLOCK*,* CRY1*,* CRY2*,* CSNK1A1*,* CSNK1D*,* MTNR1A*,* MTNR1B*,* NR1D1*,* NR1D2*,* PER1*,* PER2*,* PER3*,* RORA*,* RORB*,* RORC*,* RXRA*,* RXRB*,* TIMELESS*, and *TIPIN* (Table 1) and used the Geneious (version 6.1.7, Biomatters, Auckland, NZ) databank search function to search GenBank for sequences of each clock gene across all species. We extracted the coding sequence of each clock gene in a total of 68 mammal species, excluding humans, and used the Geneious plug‐in Phobos (Mayer, Christoph, Phobos 3.3.11, 2006–2010) to search for tandem repeats. We defined our search criteria to locate repeat units that were 3 bp long and ≥9 bp (3 units) in length. Once repeats were located, we extracted information regarding the total repeat length, percentage perfection (purity), repeat unit type (e.g., CAG or polyglutamine) and the sequence of the repeat, and calculated metrics of repeat abundance and purity across all mammal species at each candidate clock gene. We estimated the total number of repeats found, the total number of pure (i.e., 100% perfection) repeats, the total number of repeats over 5 units (15 bp) long, the total number of pure repeats over 5 units long, and the species for which the longest repeats were observed at each gene. We also explored the relationship between repeat length and repeat purity of cTNRs across the candidate genes we surveyed in mammals by conducting a Spearman's rank correlation in R (R Core Team 2016).

**Table 1:** Characterization of clock gene coding trinucleotide repeats (cTNRs) in mammal species

Gene name	Gene abbreviation	Total number of repeats found (across all species)	Total number of pure repeats found (across all species)/percentage of total repeats found	Total number of repeats found above 5 units in length/percentage of total repeats found	Total number of pure repeats found over 5 units in length/percentage of total repeats found larger than 5 units in length	Species with longest repeat found/number of repeat units
Aralkylamine N‐acetyltransferase	*AANAT*	1	1/100%	0/0%	0/0%	N/A
Aryl hydrocarbon receptor nuclear translocator‐like	*ARNTL*	49	49/100%	1/2.0%	1/100%	Long‐tailed chinchilla/5
Aryl hydrocarbon receptor nuclear translocator‐like 2	*ARNTL2*	70	70/100%	1/1.4%	1/100%	Pig/5
Clock circadian regulator	*CLOCK*	176	143/81.3%	70/39.8%	37/52.9%	Chinese hamster/26
Cryptochrome circadian clock 1	*CRY1*	33	30/90.9%	3/9.1%	0/0%	Elephant shark/5 European shrew/5 Platypus/5
Cryptochrome circadian clock 2	*CRY2*	82	77/93.9%	8/9.8%	3/37.5%	Southern white rhinoceros/12
Casein kinase 1, alpha 1	*CSNK1A1*	53	53/100%	0/0%	0/0%	N/A
Casein kinase 1, delta	*CSNK1D*	62	61/98.4%	1/1.6%	0/0%	Star‐nosed mole/7
Melatonin receptor 1A	*MTNR1A*	68	64/94.1%	4/5.88%	0/0%	Big brown bat/6
Melatonin receptor 1B	*MTNR1B*	71	71/100%	0/0%	0/0%	N/A
Nuclear receptor subfamily 1, group D, member 1	*NR1D1*	315	282/89.5%	49/15.6%	17/34.7%	Chinese hamster/10 Prairie deer mouse/10
Nuclear receptor subfamily 1, group D, member 2	*NR1D2*	49	48/98%	1/2.0%	0/0%	Platypus/10
Period circadian clock 1	*PER1*	486	449/92.4%	63/13.0%	28/44.4%	Golden hamster/30
Period circadian clock 2	*PER2*	168	125/74.4%	44/26.2%	1/2.3%	44 repeats representing 38 species/5
Period circadian clock 3	*PER3*	130	114/87.7%	16/12.3%	0/0%	Prairie vole/7
RAR‐related orphan receptor A	*RORA*	106	105/99.1%	`1/0.9%	0/0%	Lesser Egyptian jerboa/5
RAR‐related orphan receptor B	*RORB*	165	95/57.6%	70/42.4%	0/0%	70 repeats representing 48 species/5
RAR‐related orphan receptor C	*RORC*	103	71/68.9%	52/50.5%	20/38.5%	Little brown bat/19
Retinoid X receptor, alpha	*RXRA*	141	140/99.3%	2/1.4%	1/50%	European shrew/5 European shrew/5
Retinoid X receptor, beta	*RXRB*	167	156/93.4%	48/28.7%	37/77.1%	American pika/11 Rabbit/11
Timeless circadian clock	*TIMELESS*	521	397/76.2%	139/26.7%	15/10.8%	Star‐nosed mole/18 Platypus/18
TIMELESS interacting protein	*TIPIN*	101	101/100%	1/1.0%	1/100%	Domestic ferret/5

### 2.2 Study systems for investigating selection

To evaluate whether we could detect signatures of selection at candidate clock genes in natural systems, we assessed three pairs of congeneric species: Canada lynx and bobcat (*Lynx canadensis* and *L. rufus*), northern and southern flying squirrel (*Glaucomys sabrinus* and *G. volans*), and white‐footed and deer mouse (*Peromyscus leucopus* and *P. maniculatus*). Each of these species pairs had a northern distributed species and a southern congener that is expanding northwards and increasing range overlap with its sister species. Although all of these species are widely distributed and exhibit high rates of intraspecific gene flow (e.g., Garroway, Bowman, Holloway, Malcolm, & Wilson, 2011; McKay, 2016; Row et al., 2012), both theoretical (Charlesworth, Nordborg, & Charlesworth, 1997) and empirical (DeFaveri et al., 2013) studies support the prediction that selection can maintain adaptive divergence at critical loci despite the rest of the genome being homogenized via gene flow. Thus, the evolutionary histories and distributional patterns of these species pairs provide a good opportunity to survey candidate genes associated with climate change in non‐model organisms.

### 2.3 Sample collection and strategy

**Figure 1:**
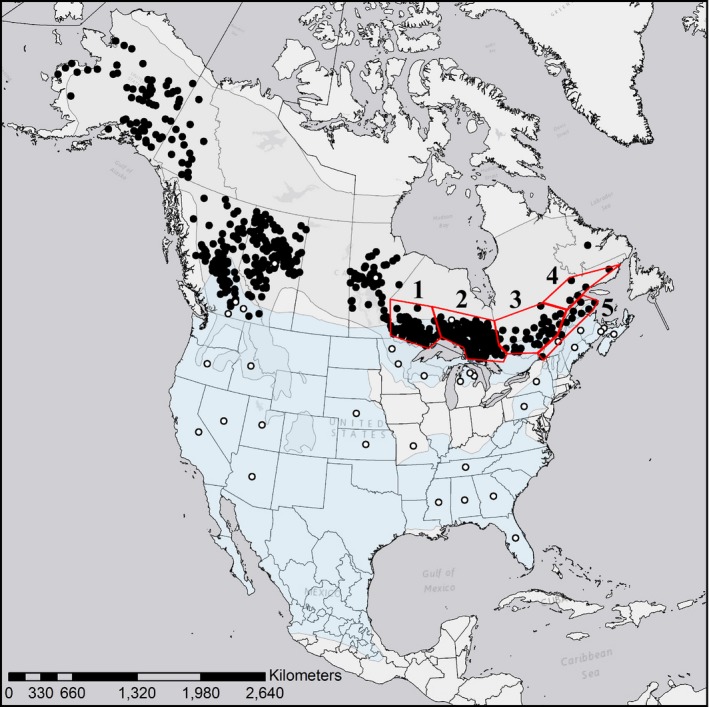
Locations of trapping sites of Canada lynx (*Lynx canadensis*, black circles) and bobcats (*Lynx rufus*, white circles) across North America. Sample coordinates of lynx represent the centroids of trapping units. Lynx samples were grouped corresponding to provincial/state boundaries, with the exception of Ontario and Quebec, Canada, which were further subdivided. Subdivision of these provinces are represented by red polygons (1 = “Ontario east,” 2 = “Ontario west,” 3 = “Quebec south,” 4 = “Quebec north,” 5 = “Quebec south of the St. Lawrence River”). Coordinates for bobcats represent the centroids of counties (USA), trap lines (Canada), or states/provinces when finer resolution spatial data were not available. The ranges of lynx and bobcat are represented in gray and blue, respectively

The spatial scale of sampling for each species pair varied, and samples were obtained from a variety of sources. The Canada lynx and bobcat analysis was continental, incorporating the entire range of both species (Figure 1), and the area of range overlap at the southern extent of Canada (Koen, Bowman, Lalor, & Wilson, 2014). Hide samples (2.5 × 2.5 mm) of legally trapped individuals were collected from the North American Fur Auction. Two sampling strategies were used. First, to test for interspecific differences, three Canada lynx samples were selected from each Canadian province and territory (excluding Nunavut), and Alaska, USA, to obtain an even representation of individuals across their geographic range (*N* = 38, Table 2). Similarly, approximately three bobcat samples were selected from across the United States representing each of the genetic clusters identified by Reding, Bronikowski, Johnson, and Clark (2012). An additional three bobcat samples were selected from each of the Canadian provinces where there was a harvest for bobcat (*N* = 52, Table 2). Second, to test for signatures of selection within Canada lynx, 1,791 lynx samples were collected and genotyped from Alaska (*N* = 89), Yukon (*N* = 28), British Columbia (*N* = 193), Alberta (*N* = 109), Manitoba (*N* = 155), Ontario (*N* = 746), Quebec (*N* = 461), and Labrador (*N* = 10). The samples used in this study were a subset of those used by Koen, Bowman, Lalor et al. (2014), and Koen, Bowman, Murray, and Wilson (2014) with the addition of samples from the western portion of the lynx range (Alaska, Yukon, Alberta, and additional British Columbia samples) (Figure 1).

**Table 2:** Sample size and location of sampling of bobcat (*Lynx rufus*) and Canada lynx (*Lynx canadensis*) to test for interspecific allelic differentiation of trinucleotide repeats at the *NR1D1* gene between the northern and southern evolved closely related species

Species	Location	Sample size
Bobcat (*Lynx rufus*)	British Columbia	3
	Alberta	2
	Manitoba	3
	Ontario	3
	Quebec	3
	New Brunswick	3
	Nova Scotia	3
	Costal Oregon^a^	3
	California^a^	3
	“western” (Nevada, Idaho, Utah, Arizona)^a^	4
	“central” (Kansas, Missouri, Nebraska)^a^	3
	“southern” (Tennessee, Alabama, Georgia)^a^	3
	“northern” (Minnesota, Wisconsin)^a^	3
	Michigan lower peninsula^a^	3
	Florida^a^	3
	Pennsylvania^a^	3
	New England^a^	3
	Total	52
Canada lynx (*Lynx canadensis*)	Alaska	3
	Yukon	3
	Northwest Territories	3
	British Columbia	3
	Alberta	5
	Saskatchewan	3
	Manitoba	3
	Ontario	3
	Quebec	3
	Labrador	3
	New Brunswick	3
	Newfoundland	3
	Total	38

a These groupings represent the sampling of genetically differentiated clusters of bobcat identified by Reding et al. (2012) across the USA.

**Figure 2:**
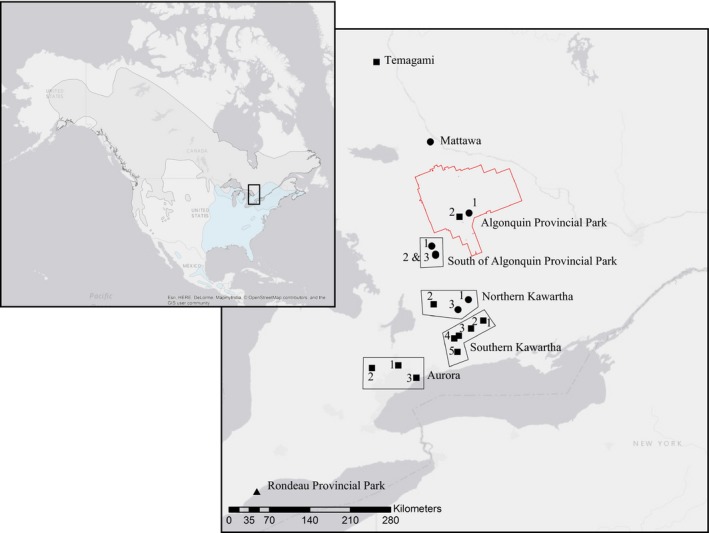
Locations of sample sites of northern flying squirrels (*Glaucomys sabrinus*) and southern flying squirrels (*Glaucomys volans*) in Ontario, Canada. Shapes of points represent the species that was trapped at each site (northern flying squirrels, southern flying squirrels, and both species represented by squares, triangles, and circles, respectively). Outlined in red is the perimeter of Algonquin Provincial Park. The inset map in the top left corner shows an overview of the sampling area within North America with the ranges of northern and southern flying squirrels shown in gray and blue, respectively. Sampling site labels correspond to sample sizes in Table 3, and site‐specific groupings are outlined by polygons

The historical range of the northern flying squirrel encompasses the coniferous and mixed coniferous forests of North America, including most of Canada and Alaska, as well as south into the United States in association with boreal remnant mountaintop habitats in both the east and the west (Linzey & NatureServe, 2008; Figure 2). The southern flying squirrel inhabits the temperate forests of eastern North America, with a historical northern range boundary at approximately 45°N latitude (Figure 2), which was estimated to be expanding by as much as 22 km/year due to warmer winters and thereby increasing overlap of this species with the range of the northern flying squirrel (Bowman, Holloway, Malcolm, Middel, & Wilson, 2005; Garroway et al., 2011). Our flying squirrel comparison was conducted on a regional scale, concentrating on an approximately 700‐km north–south transect between 42.5 and 47.2°N in Ontario, Canada, and encompassing the transition between temperate and boreal forests in the region (Bowman et al., 2005). Northern and southern flying squirrels’ ear tissue and hair samples were obtained from individuals live‐trapped between 2005 and 2010 at 19 unique trapping sites (see Bowman et al., 2005 for capture methods; Figure 2). Many trapping sites yielded individuals from both species; however, there were 11 and one sites where only northern and southern flying squirrels were trapped, respectively. Overall, 118 samples of northern and 206 samples of southern flying squirrels were genotyped along the 700‐km transect (Table 3). Some of these samples were the same as those used by Garroway et al. (2010, 2011).

**Table 3:** Sampling site coordinates and sample size of northern flying squirrels (*Glaucomys sabrinus*) and southern flying squirrels (*Glaucomys volans*) for analyses designed toward the detection of selection at the *CLOCK* exonic trinucelotide repeat motif. Sampling sites are consistent with those labeled in Figure 2. Coordinates reflect the centroid of the trapping area for each site

Species	Region	Site (Figure 2)	Latitude/longitude of site	Sample size
Northern flying squirrel (*Glaucomys sabrinus*)	Temagami		47.25/−79.76	6
	Mattawa		46.40/−78.92	5
	Algonquin Provincial Park	1	45.63/−78.32	11
		2	45.59/−78.83	14
		Algonquin Provincial Park Total		25
	South of Algonquin Provincial Park	1	45.27/−78.90	2
		2	45.18/−78.84	9
		3	45.17/−78.84	7
		South of Algonquin Provincial Park Total		18
	Northern Kawartha	1	44.68/−78.33	31
		2	44.63/−78.87	4
		3	44.57/−78.49	6
		Northern Kawartha Total		41
	Southern Kawartha	1	44.45/−78.10	2
		2	44.36/−78.29	1
		3	44.28/−78.48	3
		4	44.25/−78.55	3
		5	44.10/−78.50	6
		Southern Kawartha Total		15
	Aurora	1	43.95/−79.42	5
		2	43.92/−79.83	2
		3	43.81/−79.14	1
		Aurora Total		8
	Northern flying squirrel total			118
Southern flying squirrel (*Glaucomys volans*)	Mattawa		46.40/−78.92	4
	Algonquin Provincial Park		45.63/−78.32	2
	South of Algonquin Provincial Park	1	45.27/−78.90	1
		2	45.18/−78.84	6
		3	45.17/−78.84	27
		South of Algonquin Provincial Park Total		34
	Northern Kawartha	1	44.68/−78.33	44
		3	44.57/−78.49	118
		Northern Kawartha Total		162
	Rondeau Provincial Park		42.52/−78.84	4
	Southern flying squirrel total			206

**Figure 3:**
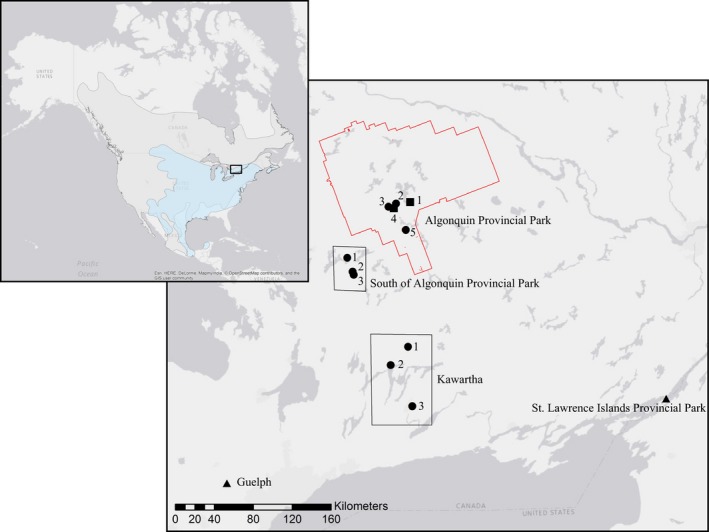
Locations of sample sites for deer mice (*Peromyscus maniculatus*) and white‐footed mice (*Peromyscus leucopus*) in Ontario, Canada. Shapes of points represent the species that was trapped at each site (white‐footed mice, deer mice, and both species represented by triangles, squares, and circles, respectively). Outlined in red is the perimeter of Algonquin Provincial Park. The insert in the top left corner shows an overview of the sampling area within North America with the ranges of white‐footed and deer mice in blue and gray, respectively. Sampling site labels correspond to sample sizes in Table 4, and site‐specific groupings are outlined by polygons

The ranges of both the white‐footed and deer mouse are large, with the white‐footed mouse existing largely in the eastern and central United States and adjoining portions of southern Canada, as well as southward into southern Mexico (Linzey, Matson, & Timm, 2008; Figure 3). The deer mouse range is also transcontinental, spanning from southern Yukon through most of the Canadian provinces, the United States (excluding the southeastern costal states) and north and central Mexico (Linzey, 2008; Figure 3). Ear punches or tail clippings of 172 white‐footed and 290 deer mice were sampled in Ontario, Canada, from individuals live‐trapped at 13 sites between Algonquin Provincial Park and the city of Guelph, Ontario during 2009–2013 (Figure 3). Trapping sites were generally species specific, with white‐footed and deer mice trapped at eight and seven sites, respectively (Table 4).

**Table 4:** Sampling site coordinates and sample size of white‐footed mice (*Peromyscus leucopus*) and deer mice (*Peromyscus maniculatus*) for analyses designed toward the detection of selection at the *PER1* exonic trinucelotide repeat motif. Sampling sites are consistent with those labeled in Figure 3. Coordinates reflect the centroid of the trapping area for each site. In bold is the sampling site where a sufficient sample size of both species was obtained

Species	Region	Site (Figure 3)	Latitude/longitude of site	Sample size
White‐footed mouse (*Peromyscus leucopus)*	Algonquin Provincial Park	2	45.62/−78.35	1
		3	45.60/−78.52	2
		5	45.45/−78.36	2
		Algonquin Provincial Park Total		5
	South of Algonquin Provincial Park	1	45.27/−78.90	11
		**2**	**45.18/−78.85**	**17**
		3	45.16/−78.84	42
		South of Algonquin Provincial Park Total		70
	Kawartha	1	44.69/−78.34	19
		2	44.57/−78.50	21
		3	44.30/−78.30	2
		Kawartha Total		42
	St. Lawrence Islands National Park		44.35/−75.96	40
	Guelph		43.79/−80.01	15
	White‐footed mice total			172
Deer mice (*Peromyscus maniculatus*)	Algonquin Provincial Park	1	45.63/−78.32	24
		2	45.62/−78.35	42
		3	45.60/−78.52	133
		4	45.59/−78.47	18
		5	45.45/−78.36	35
		Algonquin Provincial Park Total		252
	South of Algonquin Provincial Park	1	45.27/−78.90	**5**
		**2**	**45.18/−78.85**	25
		3	45.16/−78.84	**3**
		South of Algonquin Provincial Park Total		**33**
	Kawartha	1	44.69/−78.34	2
		2	44.57/−78.50	1
		3	44.30/−78.30	2
		Kawartha Total		5
	Deer mice total			290

### 2.4 DNA extraction

As both the *Lynx* and *Glaucomys* samples were extracted for prior work, the *Peromyscus* samples were the only samples that required DNA extraction (described in Appendix S1).

### 2.5 Neutral microsatellite marker datasets

For Canada lynx, we used an existing dataset of 14 neutral microsatellite loci (Fca031, Fca035, Fca077, Fca090, Fca096, Fca441, Fca391, Fca559, Lc106, Lc109, Lc110, Lc111, Lc118). These loci were also used by Koen, Bowman, Lalor, et al. (2014) and Koen, Bowman, Murray, et al. (2014) and were a subset of the loci genotyped by Row et al. (2012). For northern and southern flying squirrels, we used an existing dataset of seven neutral microsatellite loci (GS8, GS10, Pvol41, Pvol74, PvolE6, SFS3, and SFS15) generated by Garroway et al. (2010, 2011). For white‐footed and deer mice, we amplified five neutral microsatellite loci (PML01, PML03, PML04, PML11, PML12; reaction conditions and amplification parameters described in Appendix S2).

### 2.6 Selection, amplification, and genetic profiling of candidate clock gene fragments

The candidate gene fragments amplified for Canada lynx and bobcat, northern and southern flying squirrels, and white‐footed and deer mice were nuclear receptor Rev‐erbα (*NR1D1*), *CLOCK*, and *PER1*, respectively. The *NR1D1* gene is a nuclear receptor that links circadian rhythms to transcriptional control of metabolic pathways and has been documented to play an important role in establishing and maintaining circadian body temperature rhythms of cold tolerance (Everett & Lazar, 2014; Gerhart‐Hines et al., 2013). The *CLOCK* gene is a critical component of the circadian pathway, and the polyglutamine (PolyQ) motif within the *CLOCK* gene has been shown to play a role in regulating gene transcription (Darlington et al., 1998) and altering the corresponding circadian phenotype (Vitaterna et al., 1994). Specifically, Vitaterna et al. (2006) experimentally showed that mutations in the *CLOCK* gene PolyQ cTNR reduced the amplitude of the circadian pacemaker in mice, thereby effectively increasing the efficiency with which mice can synchronize to external light cues. The *PER1* gene is a light‐sensitive core component of the circadian clock (Hunt & Sassone‐Corsi, 2007), with variability in expression allowing for entrainment of circadian rhythms in synchrony with the external environment (Yamamoto et al., 2004). Importantly, alterations in expression levels have been shown to affect phenotype (Bae et al., 2001). For example, *PER1*‐deficient mice have shown impairment in survival‐related behaviors including nest building, habituation to other mice, and exploratory behavior (Bechstein et al., 2014).

Primers were designed for each cTNR using sequences of closely related model organisms in GenBank, specifically, cat (*Felis catus*) for Canada lynx and bobcat*,* house mouse (*Mus musculus*) for northern and southern flying squirrels, and human (*Homo sapiens*), house mouse *(Mus musculus*), and Norway rat (*Rattus norvegicus*) for white‐footed and deer mice (GenBank accession Nos. 101094802, NM_007715, AB002107, AF022992, and AY903228, respectively). Primers were designed in Geneious version 6.1.7 (Biomatters, Auckland, NZ) and optimized on a set of control samples for each species. Primer sequences, amplification conditions, reaction parameters, and genetic profiling of all candidate clock gene cTNRs are described inAppendices  S3 and S4.

### 2.7 Analyses for signatures of selection

Initially, genotype distributions were assessed for all species to determine whether private alleles occurred in either species of each pair, where private cTNR alleles may indicate the differential evolution of or selection on cTNR alleles in northern versus southern closely related species. Bobcat samples were excluded from the remainder of the analyses due to low sample sizes preventing intraspecific analyses for selection in bobcats.

We used GenAlEx version 6.5 (Peakall & Smouse, 2006, 2012) to calculate allele frequencies, and observed (H_O_) and expected (H_E_) heterozygosity counts. We used Genepop version 4.2 (Raymond & Rousset, 1995; Rousset, 2008) to conduct Hardy–Weinberg exact tests (HWE) on all species at both neutral microsatellites and each candidate cTNR locus, and applied a Bonferroni correction to these tests to correct for multiple pairwise comparisons. As one of the assumptions of HWE is the absence of selection, deviations from HWE of cTNR, but not neutral loci, would indicate a potential for the influence of selection on the cTNR locus. Genepop version 4.2 was also used to calculate genetic differentiation (*F*_ST_) between all population pairs at both neutral microsatellites and candidate cTNR loci. We also calculated the mean *F*_ST_ and standard error for each neutral microsatellite and cTNR dataset and compared mean neutral vs. cTNR *F*_ST_ within each species. Contrasting patterns of genetic differentiation at presumably neutral microsatellites versus putatively adaptive cTNRs would suggest that differential mechanisms are influencing neutral versus cTNR loci. For example, greater divergence at neutral compared to cTNR loci suggests that selection is favoring similar alleles across populations that do not experience high rates of gene flow. Alternatively, lower neutral genetic differentiation compared to cTNR loci suggests that selection is favoring different alleles across the examined distribution of the species despite ongoing gene flow between populations.

Population designations were determined differently for each species. Canada lynx are considered nearly panmictic across their range (Row et al., 2012), and we generally assessed each sampled province or territory separately. We subdivided the Ontario and Quebec groups according to evidence of subtle genetic structure that has been identified for lynx (Row et al., 2014; Koen, Bowman, & Wilson, 2015; Figure 1). Ontario was divided into two groups; “Ontario east” (*N* = 552) and “Ontario west” (*N* = 194) representing individuals sampled east and west of longitude −88.1, respectively (see Row et al., 2014). Quebec lynx were divided into three groups based on administrative units called Unité de Gestion des Animaux à Fourrure (UGAF). Individuals harvested within UGAFs between 1–55, 57–70, and 70–90 were grouped into the categories “Quebec south” (*N* = 261), “Quebec north” (*N* = 45), and “Quebec south of the St. Lawrence River” (*N* = 155), respectively. This division was based on a gap in sampling of lynx in UGAF 56, and the genetic structure of lynx south of the St. Lawrence River (Koen et al., 2015).

For northern and southern flying squirrels, we generally evaluated each trapping site separately, except where trapping sites were in close proximity to one another, in which case sites were grouped to increase sample sizes. Thus, for northern flying squirrels, we grouped all sites within Algonquin Provincial Park (two sites; *N* = 25), all sites located just south of Algonquin Provincial Park (three sites; *N* = 18), all sites within the “northern Kawartha” region (three sites; *N* = 41), all sites within the “southern Kawartha” region (five sites; *N* = 15), and all sites within the Aurora region (three sites; *N* = 8) (Figure 2). All other sites were evaluated separately (Table 3). For southern flying squirrels, we grouped all sites just south of Algonquin Provincial Park (three sites; *N* = 27), keeping all other sites independent for analysis (Figure 2, Table 3).

For white‐footed and deer mice, we conducted tests on two separate groupings of samples. For deer mice, we first conducted a large‐scale test by combining all sampling sites within Algonquin Provincial Park (five sites; *N* = 252), sites located just south of Algonquin Provincial Park (three sites; *N* = 33), and sites within the Kawartha region (three sites; *N* = 5) (Figure 3). We also conducted a small‐scale test at the site‐specific level to rule out microgeographic structure or possible Wahlund effects. If our results are confounded by a Wahlund effect, we would expect that sampling within a smaller geographic scale would alleviate deviations from HWE as we are more confident that we are only sampling from a single breeding population. In this test, we considered each of the five sites within Algonquin Provincial Park separately, keeping all other groupings the same for analyses due to lower sample sizes (Figure 3, Table 4). For white‐footed mice, we also conducted both large‐ and small‐scale tests. For the large‐scale analysis, we grouped all sampling sites within Algonquin Provincial Park (three sites; *N* = 5), sites located just south of Algonquin Provincial Park (three sites; *N* = 70), and sites within the Kawartha region (three sites; *N* = 42), evaluating the St. Lawrence Islands National Park and Guelph sampling sites separately (Figure 3). The small‐scale analysis considered two of the three sites within the Kawartha region (site 3 was removed due to a low sample size), and the three sites south of Algonquin Provincial Park separately, keeping all other groupings the same for analyses (Figure 3, Table 4).

We tested for evidence of selection using a coalescent‐based approach (Beaumont & Nichols, 1996) implemented in LOSITAN (Antao, Lopes, Lopes, Beja‐Pereira, & Luikart, 2008), a software platform used to detect signatures of selection based on the distribution of *F*_ST_ as a function of heterozygosity. We calculated the “neutral” mean *F*_ST_, where we first ran a simulation to remove potentially selected loci prior to computing the initial mean *F*_ST_, upon which putative adaptive loci were identified. We also selected the option to “force mean *F*_ST_,” in which LOSITAN will attempt to approximate a more precise *F*_ST_ by running a bisection over repeated simulations. We ran 50,000 simulations at a 95% confidence interval and selected a stepwise mutation model, which is commonly used to describe STR markers (Antao et al., 2008; Fan & Chu, 2007). All other parameters were left at the recommended default settings. In cases where we identified a signature of selection at any locus, two additional independent tests were conducted on the same dataset for confirmation (i.e., a true signature of selection would be expected to persist in 3/3 tests). We also removed populations iteratively with replacement from each analysis to assess the sensitivity of our results to the exclusion of individual populations.

## 3 RESULTS

### 3.1 Characterization of candidate clock genes in mammal species

In general, cTNR repeats within the coding regions of our candidate clock genes were relatively abundant (Table 1). The clock genes with the largest total number of repeats were *CLOCK*,* NR1D1*,* PER1*, and *TIMELESS*, with 176, 315, 486, and 521 total repeats across all mammal species, respectively. The majority of repeats identified within each candidate clock gene were small; repeats 3–4 units in length (9–12 bp) made up 70%–100% of the repeats found within each clock gene, with few exceptions. The genes *CLOCK*,* RORB*, and *RORC* had the greatest abundance of large repeats, with repeats >5 units (15 bp) comprising 39.8%, 42.4%, and 50.5% of all repeats within those genes, respectively. Interestingly, the candidate clock genes with the highest total number of observed repeats (*CLOCK*,* NR1D1*,* PER1*, and *TIMELESS*) were not necessarily the same as candidate genes that had the largest number of long repeats (≥5 units) (*CLOCK, RORB*, and *RORC*) (see Table S1 for a complete list of all sequences ≥5 units analyzed).

Across all of our surveyed genes, impurity was only observed in repeats that were ≥5 units long (15 bp). We often found that longer repeats were more impure than shorter repeats; however, these variables were not significantly correlated (*p* = .17). For example, the genes *CLOCK*, and *PER1*, which had some of the highest reported percentages of pure repeats (52.9% and 44.4%, respectively), were also the genes with a large number of long repeats (up to 26 and 30 units, respectively). Although a high percentage of pure, short repeats may explain the high overall purity of *PER1* repeats, it cannot explain the same observed pattern in the *CLOCK* gene, where approximately half of the pure repeats were ≥7 units. Additionally, several genes with only short observed repeats showed an extremely low percentage of total pure repeats. For example, the genes *PER2* and *RORB* had 44 and 70 repeats, respectively, all of which were a maximum of 5 units in length. However, only 2.3% and 0% were pure for *PER2* and *RORB*, respectively.

There was no obvious pattern explaining associations between species and repeat length. Many of the largest repeats were observed in small rodents, although several larger mammal species were also represented. The species with the largest repeat across all clock genes in this study was the Golden hamster (*Mesocricetus auratus*; 30 units, 90 bp, *PER1* gene), followed by the Chinese hamster (*Cricetulus griseus*; 26 units, 78 bp, *CLOCK* gene). Remarkably, the naked mole rat (*Heterocephalus glaber*), a blind species that does not rely on photoperiod cues, exhibited seven cTNRs throughout the genes examined in this study. The largest was a 22 unit (66 bp) repeat located in the *CLOCK* gene. Further, the purity of these long repeats in all three species was quite high given their length (96.67%, 93.59%, and 91.18% purity in the Golden hamster, Chinese hamster, and naked mole rat, respectively).

### 3.2 *Lynx* species and the *NR1D1* gene

**Figure 4:**
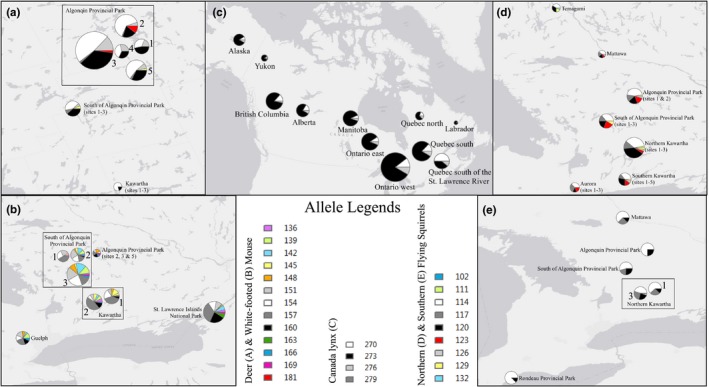
Allele frequencies for deer mice (*Peromyscus maniculatus*, a), white‐footed mice (*Peromyscus leucopus*, b), Canada lynx (*Lynx canadensis*, c), northern flying squirrels (*Glaucomys sabrinus*, d), and southern flying squirrels (*Glaucomys volans*, e) in Ontario (a,b,d,e), and across North America (c). Allele frequencies represent coding trinucleotide repeats within the candidate clock genes *PER1* (a,b), *NR1D1* (c), and *CLOCK* (d,e). Sizes of the pie charts correspond to sample sizes for each location. Rectangles represent groupings of sample sites within general areas, with specific sites indicated by numbers

A polyserine repeat motif (PolyS) was successfully amplified in Canada lynx and bobcat, and a complete segregation of nonoverlapping alleles was observed between the two species (excluding putative hybrids). Seven alleles were observed, with the smaller three occurring exclusively in bobcat and the larger four exclusively in Canada lynx, although the largest allele observed in Canada lynx was found at a very low frequency (0.008), solely in western Ontario (Figure 4c, Table 5). For the 1,791 Canada lynx samples that were analyzed for evidence of selection, none of the groups deviated from HWE at the *NR1D1* locus. However, observed homozygosity was often slightly higher than expected, and observed heterozygosity slightly lower than expected (Table S2). For the neutral marker dataset, only the Yukon population significantly deviated from HWE at the Fca441 locus (*p* = .001).

**Table 5:** Allele frequencies of the coding trinucleotide repeat marker within the *NR1D1* gene in Canada lynx (*Lynx canadensis*) sampled across North America

Population/Allele	270	273	276	279
Alaska	0.092	0.833	0.075	–
Yukon	0.093	0.833	0.074	–
British Columbia	0.134	0.834	0.032	–
Alberta	0.162	0.755	0.083	–
Manitoba	0.065	0.880	0.055	–
Ontario west	0.091	0.839	0.063	0.008
Ontario east	0.104	0.811	0.085	–
Quebec south	0.127	0.788	0.084	–
Quebec north	0.200	0.678	0.122	–
Quebec south of the St. Lawrence River	0.497	0.392	0.111	–
Labrador	0.050	0.950	–	–

**Figure 5:**
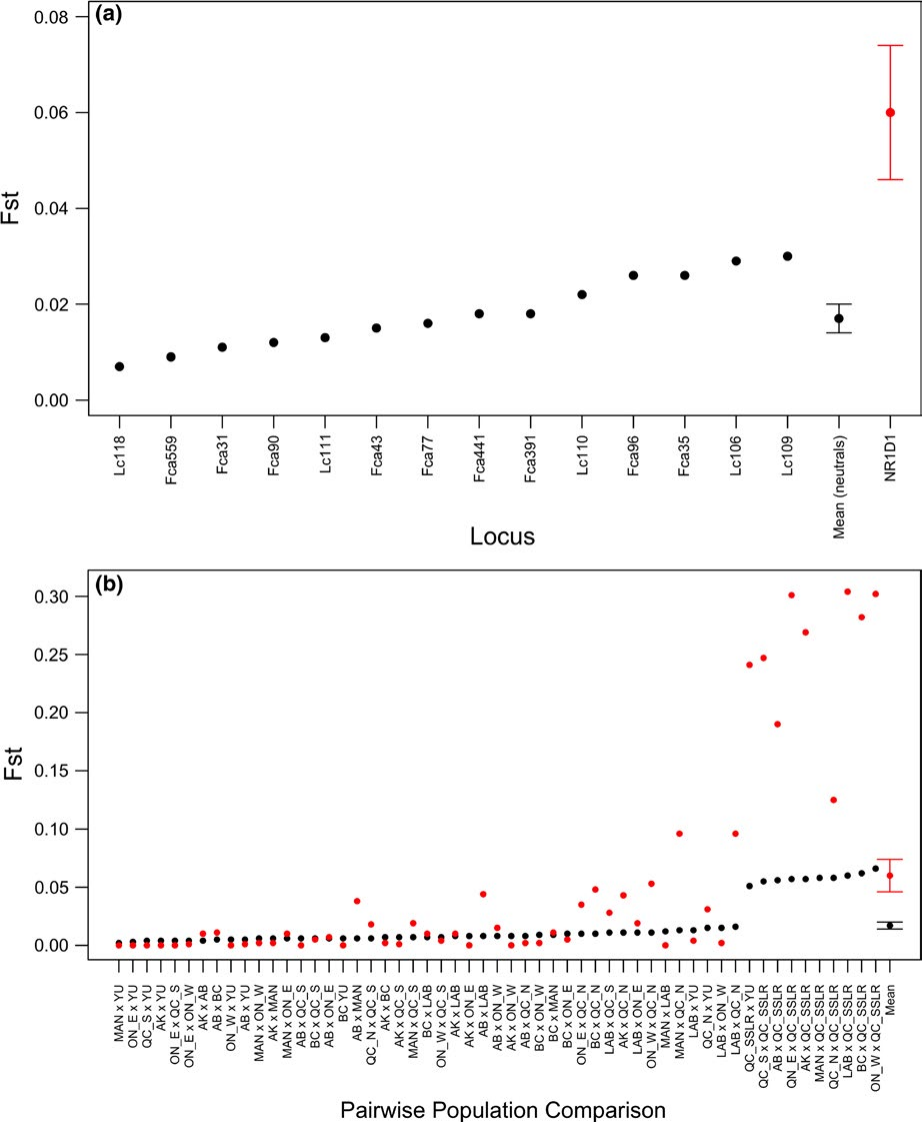
Estimates of *F*_ST_ at 14 neutral microsatellite loci (black points) and the *NR1D1* cTNR locus (red points) estimated by (a) locus and (b) pairwise population comparisons for Canada lynx (*Lynx canadensis*) sampled across Canada and Alaska. The x‐axis is ordered by increasing genetic differentiation of neutral loci, ending with estimates of mean *F*_ST_ (across loci and pairwise comparisons) for neutral and cTNR loci with standard error bars. Population abbreviations are as follows: AB (Alberta), AK (Alaska), BC (British Columbia), LAB (Labrador), MAN (Manitoba), ON_E (eastern Ontario), ON_W (western Ontario), QC_N (northern Quebec), QC_S (southern Quebec), QC_SSLR (Quebec south of the St. Lawrence River), YU (Yukon)

Average genetic differentiation (*F*_ST_) obtained from the *NR1D1* locus was substantially higher than average genetic differentiation across our set of 14 presumably neutral microsatellites (mean *F*_ST_ (*SE*) for neutral microsatellites = 0.017 (±0.003), *NR1D1* = 0.06 (±0.014); Figure 5a). Across all pairwise comparisons at both neutral markers and the *NR1D1* locus, eastern populations of lynx (i.e., Labrador, “Quebec north” and “Quebec south of the St. Lawrence River”) were the most highly differentiated groups (Figure 5b).

Once compared to a background of neutral microsatellite markers, most LOSITAN analyses detected the *NR1D1* locus as an outlier under the influence of positive selection (i.e., the *NR1D1* locus fell outside of the expected range of neutrality estimated from neutral microsatellites and within the range of F_ST_/heterozygosity that indicates positive selection). This signature of selection was only absent when the “Quebec south of the St. Lawrence River” group was removed from the analysis. We also periodically obtained a signal for positive selection at two neutral loci (Fca35 and Lc109). These neutral loci, however, straddled the edge of the positive selection range and were not consistently identified as putative outliers over multiple tests.

### 3.3 *Glaucomys* species and the *CLOCK* gene

A polyglutamine repeat motif (PolyQ) was successfully amplified in northern and southern flying squirrels. A total of nine alleles were observed at the *CLOCK* locus between the two species, which had largely overlapping allelic ranges; six of the nine alleles were found in both species. Two of the remaining three observed alleles were found solely in northern flying squirrels, and the third solely in southern flying squirrels; however, the frequencies of these three alleles were low (Table 6). The southern flying squirrel had greater allelic diversity at all neutral loci, with exception of the PvolE6 locus. In contrast, the northern flying squirrel had higher diversity in *CLOCK* alleles (an average of 5.3 alleles/site (*SE* = 0.42) in northern flying squirrel versus 3.3 alleles/site (*SE* = 0.76) in southern flying squirrel). The most common *CLOCK* allele was the same in both species; however, frequencies of this allele were slightly higher in the southern flying squirrel (Table 6). In the northern flying squirrel, the most northern trapping site (Temagami) was the only site in which the largest *CLOCK* allele was found. Temagami also showed the highest frequency of the second largest *CLOCK* allele (allele frequency of 0.167 versus 0.013–0.083 across all other sites in both species). In the southern flying squirrel, one of the northern Kawartha sites (site 3) had higher genetic variability than all other sites at the *CLOCK* locus (seven alleles vs. a maximum of three alleles across all other sites); however, many of the alleles present at this site were found at low frequencies (Table 6). Alternatively, this may be an artifact of sample size (*N* = 118 for northern Kawartha Site 3 vs. a maximum of *N* = 44 across all other sites).

**Table 6:** Allele frequencies of the coding trinucleotide repeat marker within the *CLOCK* gene in northern flying squirrels (*Glaucomys sabrinus*) and southern flying squirrels (*Glaucomys volans*) sampled in Ontario, Canada

Species	Population/allele	102	111	114	117	120	123	126	129	132
Northern flying squirrel	Temagami	–	–	0.417	–	0.333	–	–	0.167	0.083
	Mattawa	–	–	0.400	0.200	0.200	0.100	0.100	–	–
	Algonquin Provincial Park	–	0.024	0.452	0.143	0.190	0.143	0.048	–	–
	South of Algonquin Provincial Park	–	–	0.361	0.139	0.194	0.222	–	0.083	–
	Northern Kawartha	–	0.027	0.365	0.122	0.392	0.041	0.027	0.027	–
	Southern Kawartha	–	–	0.357	0.107	0.286	0.143	0.071	0.036	–
	Aurora	–	–	0.375	0.313	0.125	0.188	–	–	–
Southern flying squirrel	Mattawa	–	–	0.625	0.250	0.125	–	–	–	–
	Algonquin Provincial Park	–	–	0.750	–	0.250	–	–	–	–
	South of Algonquin Provincial Park	–	–	0.548	0.194	0.258	–	–	–	–
	Northern Kawartha Site 1	–	–	0.614	0.250	0.125	–	–	–	–
	Northern Kawartha Site 3	0.009	–	0.443	0.257	0.230	0.009	0.039	0.013	–
	Rondeau Provincial Park	–	–	0.875	–	0.125	–	–	–	–

Patterns of observed and expected homo‐ and heterozygosity were inconsistent across sites for both species. In some locations, common heterozygous genotypes were observed more than expected, whereas in others, the same genotypes were observed less than expected (Tables S3–S4).

Neither neutral microsatellite loci nor the *CLOCK* locus deviated from HWE in any of the northern flying squirrel sites. For the southern flying squirrel, the PvolE6 locus deviated from HWE in both northern Kawartha sites 1 and 3 (*p* < .001). Further, both the GS8 and Pvol74 loci were found to deviate from HWE at northern Kawartha Site 3 (*p* < .001).

**Figure 6:**
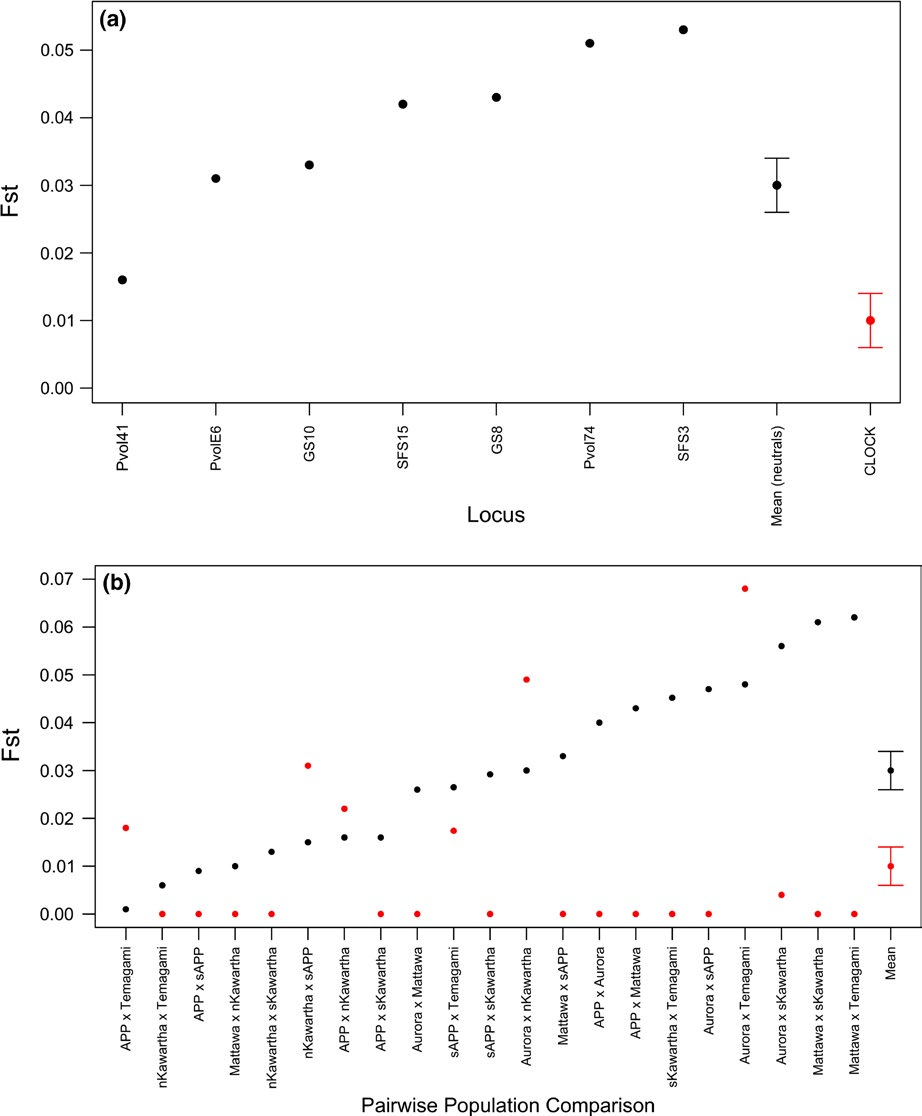
Estimates of *F*_ST_ at seven neutral microsatellite loci (black points) and the *CLOCK* cTNR locus (red points) estimated by (a) locus and (b) pairwise population comparisons for northern flying squirrels (*Glaucomys sabrinus*) sampled within Ontario, Canada. The *x*‐axis is ordered by increasing genetic differentiation of neutral loci, ending with estimates of mean *F*_ST_ (across loci and pairwise comparisons) for neutral and cTNR loci with standard error bars. Population abbreviations are as follows: APP (Algonquin Provincial Park), sAPP (south of Algonquin Provincial Park), nKawartha (northern Kawartha), sKawartha (southern Kawartha). All other population labels are written in full

**Figure 7:**
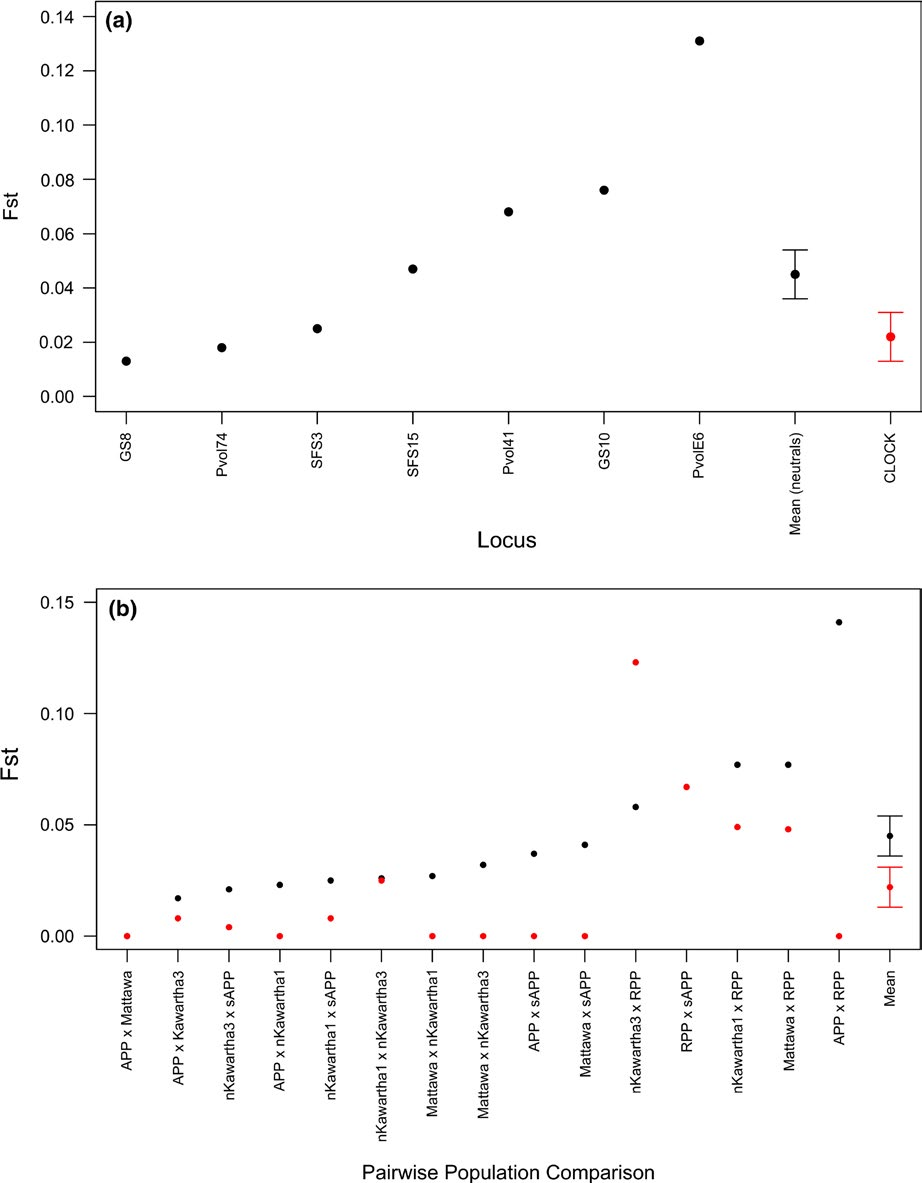
Estimates of *F*_ST_ at seven neutral microsatellite loci (black points) and the *CLOCK* cTNR locus (red points) estimated by (a) locus and (b) pairwise population comparisons for southern flying squirrels (*Glaucomys volans*) sampled within Ontario, Canada. The *x*‐axis is ordered by increasing genetic differentiation of neutral loci, ending with estimates of mean *F*_ST_ (across loci and pairwise comparisons) for neutral and cTNR loci with standard error bars. Population abbreviations are as follows: APP (Algonquin Provincial Park), sAPP (south of Algonquin Provincial Park), nKawartha (northern Kawartha), RPP (Rondeau Provincial Park). Numbers beside the “nKawartha” label represent specific sites in northern Kawartha (1,3). All other populations’ labels are written in full

Average genetic differentiation (*F*_ST_) at the *CLOCK* locus was lower than estimates obtained from our set of seven neutral microsatellites for both northern and southern flying squirrels (mean *F*_ST_ for northern [neutral microsatellites = 0.03 (±0.004), *CLOCK* = 0.01 (±0.004)], and southern flying squirrels [neutral microsatellites = 0.045 (±0.009), *CLOCK* = 0.022 (±0.009)]; Figures 6a and 7a), even though neutral genetic differentiation was quite low. For southern flying squirrels, however, the Rondeau group (the most southern site for this species) showed higher levels of genetic differentiation at neutral markers (*F*_ST_ = 0.058–0.141 for comparisons including the Rondeau group versus 0–0.041 for all other comparisons) and the *CLOCK* locus (*F*_ST_ = 0–0.123 for comparisons including the Rondeau group versus 0–0.025 for all other comparisons) across pairwise comparisons (Figure 7b).

For the northern flying squirrel, the *CLOCK* locus was identified as an outlier under balancing selection in analyses where the Aurora and Temagami groups were removed independently from the dataset. Further, the neutral microsatellites PvolE6 and Pvol41 showed signatures of balancing selection when the Aurora and southern Kawartha groups were removed, respectively. In the southern flying squirrel, evidence of balancing selection was identified at the *CLOCK* locus when the Rondeau group was removed from the analysis. A signature of positive selection was identified for the neutral microsatellite PvolE6 in several analyses including the full dataset, and when the Algonquin Provincial Park and Mattawa groups were removed. The neutral microsatellite GS8 showed a signature of balancing selection across many tests including the full dataset, and when the Mattawa, northern Kawartha Site 1, and Rondeau groups were removed. The neutral microsatellites SFS3 and SFS15 were also identified to be under balancing selection when the Algonquin Provincial Park and Rondeau groups were removed, respectively. All of the above signatures of selection were retained across three independent tests for each species.

### 3.4 *Peromyscus* species and the *PER1* gene

A polyglycine repeat motif (PolyG) was successfully amplified in white‐footed and deer mice. All neutral alleles were shared between both species; however, deer mice had greater allelic diversity than white‐footed mice across all neutral loci. In contrast, the white‐footed mouse had a higher diversity of *PER1* alleles (an average of 4.3 alleles/site (*SE* = 0.64) in deer mice versus 8.5 alleles/site (*SE* = 0.63) in white‐footed mice; measured at the small scale; Figure 4d,e; Table 7). Further, the most common *PER1* allele in the white‐footed mouse (frequencies between 0.146 and 0.452) was either much less common or completely absent from the deer mouse (frequencies between 0 and 0.088 among sites).

**Table 7:** Allele frequencies of the coding trinucleotide repeat marker within the *PER1* gene in deer mice (*Peromyscus maniculatus*) and white‐footed mice (*Peromyscus leucopus*) sampled in Ontario, Canada

Species	Population/Allele	136	139	142	145	148	151	154	157	160	163	166	169	181
Deer mouse	APP Site 1	–	–	–	–	–	0.188	0.344	–	0.469	–	–	–	–
	APP Site 2	–	–	–	–	–	0.053	0.632	0.013	0.197	–	–	–	0.105
	APP Site 3	–	–	–	–	0.005	0.127	0.471	0.015	0.358	–	0.005	–	0.020
	APP Site 4	–	–	–	–	–	0.286	0.393	–	0.321	–	–	–	–
	APP Site 5	–	–	–	0.032	–	0.081	0.532	0.032	0.323	–	–	–	–
	APP Total(sites 1–5 combined)	–	–	–	0.005	0.002	0.122	0.495	0.015	0.328	–	0.002	–	0.030
	South of APP(sites 1–3 combined)	–	–	–	0.059	–	0.059	0.412	0.088	0.382	–	–	–	–
	Kawartha(sites 1–3 combined)	–	–	–	–	–	–	0.800	–	0.200	–	–	–	–
White‐footed mouse	APP(sites 2, 3, & 5 combined)	–	–	–	0.100	0.200	–	0.100	0.200	0.200	–	0.100	0.100	–
	South of APP Site 1	0.045	–	–	0.045	–	0.227	0.273	0.409	–	–	–	–	–
	South of APP Site 2	0.031	0.094	0.125	0.063	0.031	0.250	0.188	0.156	0.031	–	0.031	–	–
	South of APP Site 3	0.024	0.110	0.146	0.024	0.085	0.195	0.220	0.146	–	–	0.024	0.024	–
	South of APP Total (sites 1–3 combined)	0.029	0.088	0.118	0.037	0.059	0.213	0.221	0.191	0.007	–	0.022	0.015	–
	Kawartha Site 1	0.026	0.053	–	0.132	0.053	0.079	0.211	0.395	0.053	–	–	–	–
	Kawartha Site 2	0.071	0.071	0.048	0.071	–	0.071	0.071	0.452	0.119	–	0.024	–	–
	Kawartha Total(sites 1–3 combined)	0.048	0.060	0.024	0.095	0.024	0.071	0.131	0.429	0.095	0.012	0.012	–	–
	St. Lawrence Islands National Park	0.039	–	0.066	0.013	0.013	0.092	0.118	0.342	0.224	0.066	–	0.026	
	Guelph	–	0.133	0.033	0.033	0.067	0.167	0.133	0.233	0.133	–	0.067	–	

For our larger‐scale analysis of deer mice, both the Algonquin Provincial Park and south of Algonquin Provincial Park groups were found to be out of HWE at the *PER1* locus, in addition to the neutral loci PML01 and PML03 in the Algonquin Provincial Park group (*p* < .0028). Excessive homozygosity of all groups at two prevalent alleles at the *PER1* locus (Tables S5–S6) suggests divergent or disruptive selection; however, it may also be a result of microgeographic structure or a Wahlund effect. We attempted to account for this by calculating HWE at the finest spatial scale of our data: the trapping sites within Algonquin Provincial Park. Three of five sites in Algonquin Provincial Park deviated from HWE at the *PER1* locus (sites 2, 3, and 5), in addition to the south of Algonquin Provincial Park group (*p* < .0012). At 3 Algonquin Provincial Park sites each, the neutral loci PML01 (sites 3, 4, and 5) and PML03 (sites 2, 3, and 5) also deviated from HWE, and PML11 was out of HWE at one site (site 2) (*p* < .0012). The persistence of deviations from HWE at the small spatial scale suggests that our results were not confounded by a Wahlund effect. Further, at the smaller scale, the signal of divergent selection at the *PER1* locus (higher observed homozygosity with fewer observed heterozygotes) was retained. For white‐footed mice, no groups significantly deviated from HWE at the *PER1* locus after Bonferroni correction at either scale; however, at the larger scale, the neutral locus PML01 was out of HWE in the south of Algonquin Provincial Park group (*p* < .0017). Additionally, no sites at either the small or large scale demonstrated excessive observed homozygosity in white‐footed mice (Tables S7–S8).

**Figure 8:**
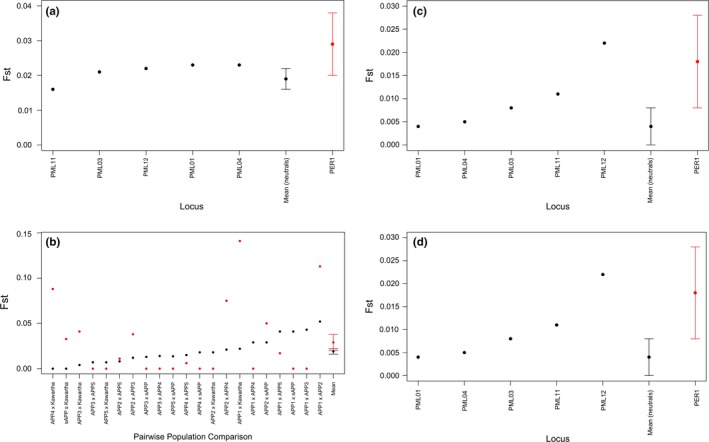
Estimates of *F*_ST_ at five neutral microsatellite loci (black points) and the *PER1* cTNR locus (red points) estimated by (a,c) locus and (b,d) pairwise population comparisons at the “trapping site‐specific” small scale (a,b), and “regional” large scale (c,d) for deer mice (*Peromyscus maniculatus*) sampled within Ontario, Canada. The *x*‐axis is ordered by increasing genetic differentiation of neutral loci, ending with estimates of mean *F*_ST_ (across loci and pairwise comparisons) for neutral and cTNR loci with standard error bars. Population abbreviations are as follows: APP (Algonquin Provincial Park), sAPP (south of Algonquin Provincial Park). Numbers beside the “APP” label represent specific sites in Algonquin Provincial Park (1–5). All other population labels are written in full

**Figure 9:**
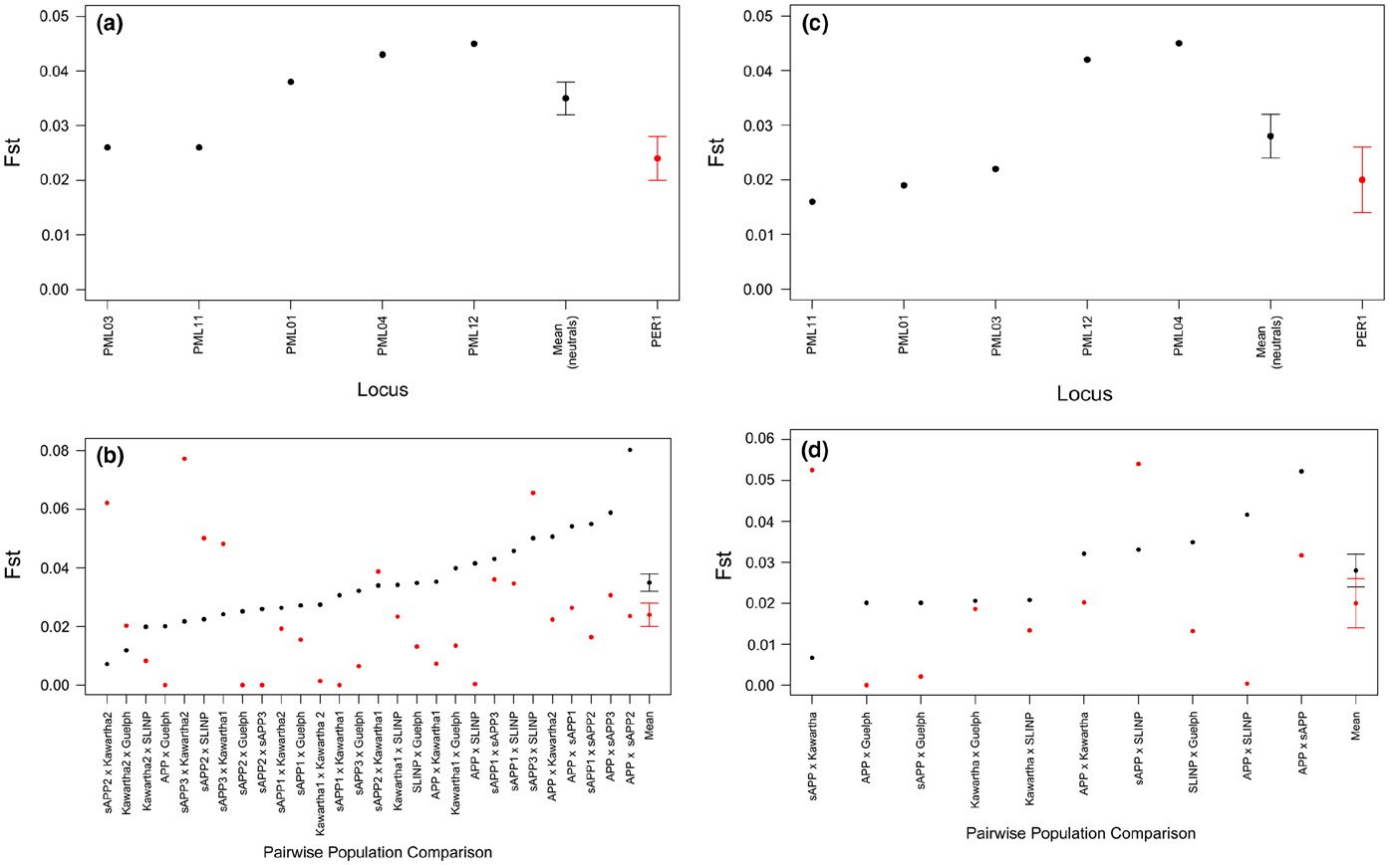
Estimates of *F*_ST_ at five neutral microsatellite loci (black points) and the *PER1* cTNR locus (red points) estimated by (a,c) locus and (b,d) pairwise population comparisons at the “trapping site‐specific” small scale (a,b), and “regional” large scale (c,d) for white‐footed mice (*Peromyscus leucopus*) sampled within Ontario, Canada. The *x*‐axis is ordered by increasing genetic differentiation of neutral loci, ending with estimates of mean *F*_ST_ (across loci and pairwise comparisons) for neutral and cTNR loci with standard error bars. Population abbreviations are as follows: APP (Algonquin Provincial Park), sAPP (south of Algonquin Provincial Park), SLINP (St. Lawrence Islands National Park). Numbers beside the “sAPP” and “Kawartha” labels represent specific sites south of Algonquin Provincial Park (1–3) and Kawartha (1–2), respectively. All other population labels are written in full

At the large scale, average genetic differentiation (*F*_ST_) for the deer mouse was higher at the *PER1* locus in comparison with our neutral microsatellite dataset (mean *F*_ST_ for neutral microsatellites = 0.004 (±0.004), and *PER1* = 0.018 (±0.01); Figure 8c). Further, while the Algonquin Provincial Park and south of Algonquin Provincial Park pairwise comparison was most genetically differentiated at neutral loci (*F*_ST_ = 0.012 for Algonquin Provincial Park and south of Algonquin Provincial Park comparison, vs. *F*_ST_ = 0 for all other comparisons), it was the least differentiated at the *PER1* locus (*F*_ST_ = 0 for Algonquin Provincial Park and south of Algonquin Provincial Park comparison, vs. *F*_ST_ = 0.023–0.033 for all other comparisons) (Figure 8d). At the smaller scale, average genetic differentiation for deer mouse was slightly greater at both neutral markers and *PER1* than was observed in the larger‐scale analysis (mean *F*_ST_ for neutral microsatellites = 0.019 (±0.003), and *PER1* = 0.029 (±0.009); Figure 8a), although standard error bars for neutral loci and the *PER1* cTNR locus overlapped. At this scale, the Algonquin Provincial Park Site 1 group was the most differentiated of all groups at neutral loci (*F*_ST_ = 0.022–0.052 for comparisons including the Algonquin Provincial Park site 1 group vs. 0–0.029 for all other comparisons; Figure 8b). For white‐footed mouse, both the large‐scale and small‐scale analyses yielded similar results; the Algonquin Provincial Park group was the most highly differentiated from other groups at neutral markers (*F*_ST_ = 0.020–0.080 and 0.020–0.052 at the small and large scales for comparisons including the Algonquin Provincial Park group vs. 0.007–0.055 and 0.007–0.035 at the small and large scales for all other comparisons, respectively; Figure 9b,d), and neutral genetic differentiation was slightly higher at the small scale (mean *F*_ST_ for neutral microsatellites at the small scale = 0.035 (±0.003), and large scale = 0.028 (±0.004); Figure 9a,c). In contrast to the deer mouse, average genetic differentiation at the *PER1* locus was lower than in neutral microsatellites at both scales [small scale: mean *F*_ST_ for neutral microsatellites = 0.035 (±0.003), and *PER1* = 0.024 (±0.004); large scale: mean *F*_ST_ for neutral microsatellites = 0.028 (±0.004), and *PER1* = 0.021 (±0.006)]; however, standard error bars for neutral loci and the *PER1* cTNR locus overlapped at the large scale (Figure 9d).

For the deer mouse at the small scale, we found a signature of positive selection at the *PER1* locus when the Algonquin Provincial Park Site 1 was removed from the analysis, but this signature was only retained in two of three independent tests, and disappeared when analyzed at the large scale. For white‐footed mouse, no signature of selection was detected at the small scale; however, a signature of balancing selection was detected for the *PER1*, locus at the large scale when the Algonquin Provincial Park group was removed from the analysis. This analysis also indicated balancing selection at the neutral microsatellite PML03 when the St. Lawrence Islands group was removed from the analysis.

## 4 DISCUSSION

### 4.1 Characterization of candidate clock genes in mammal species

We observed a number of trends with respect to the presence, abundance, and purity of cTNRs in a range of mammal species. Repeats were generally abundant across most of our candidate clock genes. Due to the overall short length, general abundance, and consistent purity of repeats that were 4 units (12 bp) or smaller, we suggest that the most promise for identifying selection will be found in repeats that surpass this threshold.

We found that longer repeats were sometimes more impure than shorter repeats; however, this trend was inconsistent across genes, and the overall relationship was not significant. Thus, the tendency for there to be a negative relationship between repeat purity and length may be tempered by variability among genes. Interestingly, impurity has been reported to significantly affect the stability of repeat structures (Pearson et al., 1998). A decrease of up to several orders of magnitude in the overall mutability of repeat fragments has been reported when impurities are present within shorter repeats, in multiple numbers, or near the center of the repeat unit (Ananda et al., 2014). This suggests that impurities within exonic repeat structures may be selected for or against. For some genes, increased mutability and variation in repeat length might favor selection for pure repeats, whereas for other genes, a more stable repeat structure might be selected for to reduce maladaptation and essentially “lock‐in” favorable geno‐ and phenotypes within an optimal functional range.

We also found that the longest repeats were in domestic rodent species, suggesting that domestics may experience elevated mutation rates in cTNRs (see also Laidlaw et al., 2007). Similarly, we found a large repeat in the naked mole rat, a blind species which does not rely on photoperiodic cues. It is possible that the expansion of cTNRs in both domestic and wild species that do not use photoperiod to cue life‐history events could be caused by the lifting of evolutionary constraints on repeat size; however, this idea remains largely unsupported. Further, the considerable purity of these long repeats supports the claim that increased purity is the mechanism by which cTNRs mutate (Ananda et al., 2014; Gemayel et al., 2012; Kruglyak et al., 1998). Thus, it is plausible that the removal of selective constraints, due to domestication or nonreliance on photoperiod, has removed the necessity for stable repeat structures to avoid maladaptation and thus facilitated the expansion of cTNRs in these species.

### 4.2 Evidence of selection in candidate clock gene cTNRs of North American mammal species

We were able to detect signatures of selection at several candidate clock genes in our range of mammal study species with varying degrees of success. Patterns of allele frequencies, allelic diversity, and/or HWE deviations from the patterns typical of neutral markers indicated that selection pressures are potentially influencing these loci in our species. Additionally, observed patterns of genetic differentiation at cTNRs were divergent from levels of differentiation observed at neutral microsatellite markers across all species, as predicted. Higher genetic structure in cTNRs (e.g., Canada lynx and deer mouse) could possibly be the result of selection favoring different alleles across the species’ distributions, despite the homogenizing effects of gene flow on neutral markers. In contrast, average genetic differentiation that is lower at candidate cTNRs than estimates obtained from neutral markers (e.g., white‐footed mouse, northern and southern flying squirrels) may reflect selective pressures that favor conserved genetic variants across the species’ distributions, despite population structure at neutral markers. Our LOSITAN analyses provided additional support for the influence of selection on the cTNR loci studied here. We found that LOSITAN analyses were sensitive to the effects of geography in each of our mammal species and were able to identify populations that contributed the most to the signature of selection detected in the cTNR locus of each species.

We found a complete divergence of alleles at the *NR1D1* locus between Canada lynx and bobcat, supporting the role of selection in the separate Pleistocene evolution of closely related species, and subsequent adaptation of Canada lynx and bobcat to more northern and southern climatic habitats, respectively. While divergence times of these species may account for the divergence of alleles at the *NR1D1* locus, the same pattern is not found in any of the neutral loci, whose allelic ranges are largely shared between species, thus supporting the role of selection in maintaining divergence at the *NR1D1* locus. Further, the higher level of pairwise genetic differentiation reflected in the *NR1D1* locus of Canada lynx is compelling evidence for selection in the face of otherwise homogenizing gene flow, represented by low levels of differentiation in neutral markers. Our adaptation‐driven hypothesis was also supported by LOSITAN, which indicated a signature of positive selection for Canada lynx at the *NR1D1* locus when the “Quebec south of the St. Lawrence River” population was included in analyses, suggesting that this southern population is largely driving the signature of selection that we detected. It should be noted, however, that neutral genetic differentiation was also highest in pairwise comparisons including the “south of the St. Lawrence River” population (Koen et al., 2015).

Although *CLOCK* alleles were largely shared between northern and southern flying squirrels, there was evidence of differentiation in allele frequencies of some of the more commonly observed alleles. This suggests that different alleles may be selectively favored over others in accordance with the differing habitats of the northern vs. southern species. In addition to the divergence of allele frequencies, the greater genetic diversity of *CLOCK* alleles found in the northern flying squirrel may allow for greater fine‐tuning capabilities in northern flying squirrel life‐history strategies to cope with the more severe seasonal changes in their northern environment. We identified the *CLOCK* gene as within the range of balancing selection in LOSITAN for both northern and southern flying squirrels when the most southern (for both species) and northern (for northern flying squirrel only) sites were removed from the analysis, suggesting that these geographically “extreme” groups were influencing the signal of selection in both species.

As in the flying squirrels, differences in allele frequency distributions of the white‐footed and deer mouse indicate potential differential selection between the two closely related species. Further, in the deer mouse, deviations from HWE and an excess of observed homozygotes at the most prevalent *PER1* alleles in both large‐ and small‐scale analyses of sites within and surrounding Algonquin Provincial Park suggest disruptive selection in this area, where small‐scale changes in environmental features (e.g., microhabitats) may drive the selection of particular alleles in slightly different environments. These results would be predicted if there were mice with predispositions to breeding at different times of the year, essentially “isolation by time” (Hendry & Day, 2005). Pairwise estimates of genetic differentiation at our set of neutral microsatellites and the *PER1* locus showed contrasting patterns for white‐footed and deer mouse, indicating that diverse processes may be influencing the two closely related species differently. We found that the northern residing species had lower allelic diversity at the *PER1* locus, contrary to our expectations that we should find higher allelic diversity to allow for greater seasonal fine‐tuning capabilities (as observed in flying squirrels). However, the sampled gradient used here is small with much of the sampled area being inhabited by both species; a wider latitudinal gradient may clarify these results. Lastly, a persistent signature of balancing selection was observed in the white‐footed mouse when the Algonquin Provincial Park site (the most northern sampled site) was removed from the LOSITAN analyses.

Combined, our set of analytical approaches was able to detect signatures of selection in the cTNRs of candidate clock genes in an array of North American mammals along latitudinal clines. Our results suggest that these techniques are useful for surveying candidate genes in non‐model species and that cTNRs are interesting markers to investigate in reference to mammalian adaptation. Further, the diversity of analyses used to detect selection in these genetic markers suggests that testing for indications of selection is best when multiple approaches are implemented that are able to detect different types of selective processes (positive, balancing, divergent, etc.). While the signatures we detected in our datasets point to the potential for adaptive differences at these coding motifs, our findings support the need for more extensive characterization of populations (e.g., assessing correlates with environmental variables).

### 4.3 Limitations on the detection of selection

The influence of sample size on the accurate detection of selection signatures is important to consider given some of our datasets. Lachance (2009) showed that sample sizes ranging from thousands to millions are needed to detect departures from HWE resulting from selection. Such sample sizes are often difficult if not impossible to obtain in wild study systems. Further, small sample sizes often misrepresent the true allele frequencies of populations, which can cause issues in downstream analyses.

In addition to sample sizes, the use of HWE to detect signatures of selection may be disadvantageous in systems experiencing directional selection, as cTNRs under the influence of directional selection are less likely to be observed at intermediate allele frequencies, which are preferable for the detection of departures from HWE (Lachance, 2009). Thus, departures from HWE may not necessarily imply selection on the marker under examination. Alternatively, background population structure can modify cTNR allele and genotype frequencies to the extent that any signature of selection is effectively masked by other mechanisms (Ennis, 2007).

Differentiation‐based outlier approaches (e.g., LOSITAN) have also received some criticism in the literature. Such approaches generally have lower power than approaches based on environmental associations (De Mita et al., 2013) as they mainly aim to detect hard selective sweeps where only one or few beneficial alleles are selected to high frequency, producing more significant patterns of differentiation between populations (Hohenlohe, Philips, & Cresko, 2010; Raquin et al., 2008). Most genes, on the other hand, act in pleiotropy (Harrisson et al., 2014), which produces more modest increases in allele frequencies over multiple loci (Hermisson & Pennings, 2005) and is less likely to affect the patterns of divergence between populations. Indeed, LOSITAN does not address the issue of non‐linearity of *F*_ST_ estimates that approach zero and, thus, is unlikely to detect low‐*F*_ST_ outliers when selection is not strong (Antao et al., 2008). In such cases, the detection of weak, polygenic selection requires much larger sample sizes than those required to detect hard sweeps.

It is also possible that cTNR motifs may not be the specific regions under selection in adaptive genes but are rather linked to other genes or regulatory elements under selection. It has been suggested that multilocus metrics of linkage disequilibrium (LD) are better suited for the detection of selection, as selection will result in LD adjacent to the selected locus (Ennis, 2007). It also has the added benefit of bearing the footprint of past selection (Lachance, 2009), which can provide important information on the genetic responses of species under past environmental change. Even if cTNRs are not the markers under selection, their increased variability and linkage to coding SNPs and regulatory elements could still be an important proxy for detecting candidate adaptive genes through haplotype profiling and analyses.

Although our approach was successful at identifying putative patterns of adaptive genetic divergence in our range of mammal species, further work is required to fully realize the mechanisms underlying the observed functional variation in these species, as it has been demonstrated that the mechanisms underlying phenotype–genotype correlations can differ between closely related species (Rosenblum, Römpler, Schöneberg, & Hoekstra, 2010). The general complexity of molecular mechanisms coupled with the pleiotropic effects of many genes argues for caution in interpretation of our results; however, we feel that we have provided support for the importance of cTNRs as targets of natural selection and adaptation in wild populations.

### 4.4 Potential adaptive importance of cTNR loci

In the recent past, there has been an advancement in empirical studies implicating climatic and environmental gradients in the generation and maintenance of adaptive genetic diversity through selection, even in the face of ongoing gene flow between populations (DeFaveri et al., 2013; Fang et al., 2013; Watanabe, Kazama, Omura, & Monaghan, 2014). Clock genes in particular have been demonstrated to be important targets of selection, as they are likely to provide a means by which species can adapt to seasonal changes or adjust to novel environments (Kondratova et al., 2010). Our utilization of a candidate gene approach allowed us to identify and target cTNRs within clock genes characterized in closely related model organisms for which functional roles have been identified. Not only did this type of approach make optimizing gene fragments easier by facilitating primer design, but it also allowed us to use prior knowledge of gene function to develop a priori hypotheses regarding the environmental and climatic factors that may be driving selection. Thus, this methodology is useful in determining cTNR‐containing genes that are good candidates for environmental association studies that correlate environmental variants with adaptive genetic variability in a spatially explicit framework (e.g., latent factor mixed models, LFMM; Frichot, Schoville, Bouchard, & François, 2013). This will lead us one step closer to being able to accurately characterize genotype–phenotype associations in wild populations.

Gene motifs, specifically cTNRs demonstrating both genetic and epigenetic characteristics (Gemayel et al., 2010, 2012), may provide high‐pace adaptive capabilities, making them ideal targets for mitigating the decline of species at risk through the identification of adaptively significant populations. A critical development in modeling a species’ natural resilience (Dawson et al., 2011) and implementing solutions (e.g., Thomas et al., 2012) is mapping and promoting environments to maintain critical standing adaptive genetic variation and the potential generation of novel adaptive alleles; cTNRs offer the potential to support both of these objectives. We present here a methodology by which we were able to identify cTNRs that are potentially the targets of natural selection in a range of mammal species, a taxonomic group underestimated in terms of vulnerability to climate change. Variance at cTNR motifs in other genes may provide a mechanism for rapid evolutionary responses to a range of other phenotypes. Thus, the abundance of cTNR repeats in functional gene classes including but not limited to clock, immunity, behavioral, morphological, and stress‐axis genes translates into a resource list of hundreds of candidate genes that can be used in the search for rapidly evolving motifs associated with adaptation in wild species.

## CONFLICT OF INTEREST

None declared.

## DATA ARCHIVING

Data for this study are available at: to be completed after manuscript is accepted for publication.
