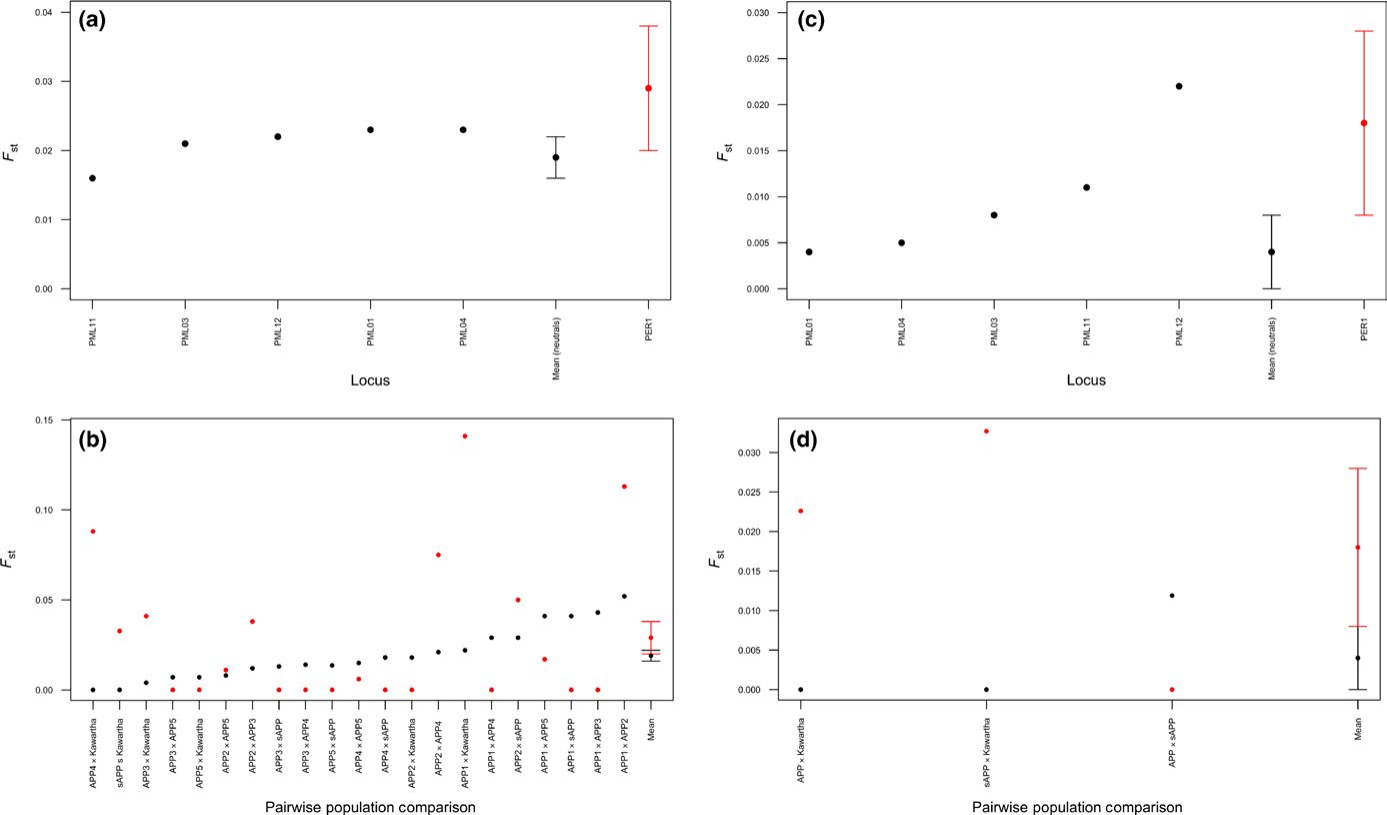# Corrigendum

**DOI:** 10.1002/ece3.3587

**Published:** 2017-11-08

Prentice, M. B., Bowman, J., Lalor, J. L., McKay, M. M., Thomson, L. A., Watt, C. M., … Wilson, P. J. (2017). Signatures of selection in mammalian clock genes with coding trinucleotide repeats: implications for studying the genomics of high‐pace adaptation. Ecology and Evolution, 7, 7254–7276

Figure 8 was mistakenly published with the top right‐hand panel duplicated in the bottom right corner of the figure, in place of the correct panel. In this corrigendum, the bottom right‐hand panel has been replaced with the correct panel.