# Supplementary material for: Signatures of selection in mammalian clock genes with coding trinucleotide repeats: Implications for studying the genomics of high‐pace adaptation
Source: Ecol Evol. 2017 Aug 8;7(18):7254–76. doi: 10.1002/ece3.3223 (PMC5606889; doi:10.1002/ece3.3223)
Supplement: Supplementary file 1 [file ECE3-7-7254-s001.docx]

APPENDIX S1: DNA extraction of *Peromyscus* samples

DNA was extracted from the tissue samples by placing each sample in lysis solution with 10ul of proteinase K (Roche, Basel, Switzerland), and subsequently placing the samples in a 65ºC water bath for 2 hours. A second 10ul aliquot of proteinase K was then added to the samples, which were then placed in a 37ºC incubator for a 24-hour period. Samples were mixed vigorously by vortexing them between each addition of proteinase K solution. DNA was extracted from the lysed samples with a Qiagen (Hilden, Germany) DNeasy Blood and Tissue kit following the manufacturer’s protocols.

APPENDIX S2: Amplification conditions of *Peromyscus* neutral microsatellites

*Peromyscus leucopus*, and *Peromyscus maniculatus* samples were amplified by polymerase chain reaction (PCR) at five di-nucleotide microsatellite loci: PML01, PML03, PML04, PML11, PML12 (Chirhart et al. 2000) using fluorescently labeled (HEX, FAM, PET, NED) primers (Appendix S5). Amplification was performed in 12-ul reaction volumes containing 1x PCR buffer, 2 mM MgCl, 0.2 mM dNTPs, 0.2 mg/ml BSA, 0.3-0.4 uM of forward and reverse primers (Appendix S5), 5 ng DNA and 0.3U of Taq polymerase (Invitrogen). PCR conditions included an initial denaturing period of 5 min at 95°C followed by 35 cycles of 94°C for 30 sec., primer-specific annealing temperature (50-58°C depending on primer pair, Appendix S5) for 1 min and 68°C followed by a final extension time of 15min at 65°C.

APPENDIX S3: Amplification and genetic profiling of candidate clock gene cTNR fragments

Amplification for Canada lynx and bobcat was conducted in a 10ul reaction containing deionized water, 1X PCR Reaction Buffer, 2 mM MgCl_2_, 0.2 mM dNTP solution, 0.2 mM Bovine Serum Albumin (BSA), 0.3 uM forward and reverse primers (forward primer labeled with the fluorescent dye 6-FAM), and 0.025U of Taq DNA Polymerase. For northern and southern flying squirrels, a total reaction volume of 10ul contained deionized water, 1X PCR Reaction Buffer, 1.5 mM MgCl_2_, 0.2 mM dNTP solution, 0.1 mM BSA, 0.3 uM forward and reverse primers (forward primer labeled with the fluorescent dye 6-FAM), and 0.04U of Taq DNA Polymerase. For white-footed and deer mice, a total reaction volume of 10ul contained deionized water, 1X PCR Reaction Buffer, 1.5 mM MgCl_2_, 0.2 mM dNTP solution, 0.25 mM BSA, 0.1 uM forward and reverse primers (forward primer labeled with the fluorescent dye HEX), and 0.025U of Taq DNA Polymerase. A total of 5ng of DNA of each sample was used for amplification across all species. All amplification reagents were acquired from Invitrogen and primers from Integrated DNA Technologies. Amplification was performed in a Bio-Rad DNA Engine Dyad and Dyad Disciple thermocycler under the conditions outlined in Appendix S4.

Prior to genotyping, all amplified samples were diluted to 1:50 with deionized water (Invitrogen). Genotyping was conducted on the Applied Biosystems (ABI) 3730 DNA Analyzer using GeneScan 500 ROX (Applied Biosystems) as a size standard for all species. Genotypes were scored manually with GeneMarker AFLP/Genotyping Software Version 1.91 (Softgenetics, Pennsylvania, USA). Scoring was conducted by two independent observers to ensure consistency of scores.

APPENDIX S4: List of functional gene amplified, location of amplified region, type of repeat, primer sequence, and thermocycler parameters for the isolation and optimization of coding trinucleotide repeats (cTNRs) within candidate clock gene fragments in Canada lynx & bobcat, northern flying squirrel & southern flying squirrel and white-footed mouse & deer mouse. Also included is the number of observed alleles and allelic range for the coding trinucleotide repeat of

each species pair.

| **Species** | **Functional Gene Amplified** | **Location of Amplified Region** | **Type of Repeat** | **Primer Sequence** | **Thermocycler Parameters** | **Number of Alleles (Allelic Range)** |
| --- | --- | --- | --- | --- | --- | --- |
| Canada lynx & bobcat | *NR1D1* | Exon 2 | Imperfect PolyS (serine) | F^1^: 5’-TAC AGT GAC AGC TCA AAT GGC-3’ | 1. 95ºC for 10 min. | 6 alleles (258-276bp) |
|  |  |  |  |  | 2. 30 cycles of: 94ºC for 30 sec. 62ºC for 1 min. 72ºC for 1 min. |  |
|  |  |  |  | R^2^: 5’-GTG ATG TTG CTG GTG CTC TT-3’ | 3. 65ºC for 15 min. |  |
| northern flying squirrel & southern flying squirrel | *CLOCK* | Exon 18/19 | Imperfect PolyQ (Glutamine) | F^1^: 5'-AGG TGG TGA CTG CCT ATC CT-3' | 1. 95ºC for 10 min. | 9 alleles (102-132bp) |
|  |  |  |  | R^2^: 5'-CTG TTC CTG GGA ACT CTG CTG-3' | 2. 30 cycles of: 94ºC for 30 sec. 60ºC for 1 min. 72ºC for 1 min. |  |
|  |  |  |  |  | 3. 65ºC for 15 min. |  |
| white-footed mouse & deer mouse | *PER1* | Exon 21 | Imperfect PolyG (Glycine) | F^1^: 5’-CCT GGC CAT TCT GAT GAC CCA C -3’ | 1. 90ºC for 5 min. | 12 alleles (136-169bp) |
|  |  |  |  |  | 2. 35 cycles of: 95ºC for 30 sec. 58ºC for 30 sec. 72ºC for 30 sec. |  |
|  |  |  |  | R^2^: 5’-CTG GGC CTC CTA CCA CCG TCA CAC-3’ | 3. 72ºC for 45 min. |  |

**F^1^ refers to the forward primer sequence; R^2^ refers to the reverse primer sequence.**

APPENDIX S5: *Peromyscus* primers used in microsatellite analysis of *Peromyscus leucopus* and *Peromyscus maniculatus*. Included are the name of the loci being amplified, fluorescent label of the primer, primer-specific annealing temperature (TA) and concentration of primer (uM) used in each 12-ul reaction.

| **Locus** | **Label** | **TA (°C)** | **Primer (uM)** |
| --- | --- | --- | --- |
| PML01 | NED | 50 | 0.4 |
| PML03 | PET | 54 | 0.4 |
| PML04 | NED | 58 | 0.3 |
| PML11 | HEX | 58 | 0.4 |
| PML12 | HEX | 58 | 0.3 |
