# Supplementary material for: Signatures of selection in mammalian clock genes with coding trinucleotide repeats: Implications for studying the genomics of high‐pace adaptation
Source: Ecol Evol. 2017 Aug 8;7(18):7254–76. doi: 10.1002/ece3.3223 (PMC5606889; doi:10.1002/ece3.3223)
Supplement: Supplementary file 3 [file ECE3-7-7254-s003.docx]

***Signatures of selection in mammalian clock genes with coding trinucleotide repeats: implications for studying the genomics of high-pace adaptation***

***Supplementary Tables Legend***

Prentice MB, Bowman J, Lalor JL, McKay MM, Thomson LA, Watt CM, McAdam AG, Murray DL, Wilson PJ

**Supplementary Tables Legend:**

***Note: supplementary tables are all contained within a single excel file. Tables can be identified by the headings “Table S1” etc., under separate tabs within the excel file. Headings for the tables describing the contents can be found below.**

**Table S1.** Complete list of all mammalian sequences (excluding humans) within GenBank for a selection of 19 clock gene coding trinucleotide repeat fragments (cTNRs) longer than 5 units in length (15 bp). Listed is the gene name, species scientific and common names, length of the repeat (bp), type of repeat unit (i.e., amino acid codon), repeat sequence and percentage purity of the repeat. Note that the sequences are of the repeat unit only and not of the entire gene.

**Table S2.** Observed (H_O_) and expected (H_E_) heterozygosity of 1,791 Canada lynx (*Lynx canadensis*) at the coding trinucleotide repeat marker within the *NR1D1* gene.

**Table S3.** Observed (H_O_) and expected (H_E_) heterozygosity of 118 northern flying squirrels (*Glaucomys sabrinus*) at the coding trinucleotide repeat marker within the *CLOCK* gene.

**Table S4.** Observed (H_O_) and expected (H_E_) heterozygosity of 206 southern flying squirrels (*Glaucomys volans*) at the coding trinucleotide repeat marker within the *CLOCK* gene.

**Table S5.** Observed (H_O_) and expected (H_E_) heterozygosity of 290 deer mice (*Peromyscus maniculatus)* at the coding trinucleotide repeat marker within the *PER1* gene measured at the small-scale.

**Table S6.** Observed (H_O_) and expected (H_E_) heterozygosity of 290 deer mice (*Peromyscus maniculatus)* at the coding trinucleotide repeat marker within the *PER1* gene measured at the large scale.

**Table S7.** Observed (H_O_) and expected (H_E_) heterozygosity of 172 white-footed mice (*Peromyscus leucopus)* at the coding trinucleotide repeat marker within the *PER1* gene measured at the small scale.

**Table S8.** Observed (H_O_) and expected (H_E_) heterozygosity of 172 white-footed mice (*Peromyscus leucopus)* at the coding trinucleotide repeat marker within the *PER1* gene measured at the large scale.
